# Correction of Dystrophin Expression in Cells From Duchenne Muscular Dystrophy Patients Through Genomic Excision of Exon 51 by Zinc Finger Nucleases

**DOI:** 10.1038/mt.2014.234

**Published:** 2015-01-13

**Authors:** David G Ousterout, Ami M Kabadi, Pratiksha I Thakore, Pablo Perez-Pinera, Matthew T Brown, William H Majoros, Timothy E Reddy, Charles A Gersbach

**Affiliations:** 1 Department of Biomedical Engineering, Duke University, Durham, North Carolina, USA; 2 Program in Computational Biology and Bioinformatics, Duke University, Durham, North Carolina, USA; 3 Center for Genomic and Computational Biology, Duke University, Durham, North Carolina, USA; 4 Department of Biostatistics and Bioinformatics, Duke University Medical Center, Durham, North Carolina, USA; 5 Department of Orthopaedic Surgery, Duke University Medical Center, Durham, North Carolina, USA

## Abstract

Duchenne muscular dystrophy (DMD) is caused by genetic mutations that result in the absence of dystrophin protein expression. Oligonucleotide-induced exon skipping can restore the dystrophin reading frame and protein production. However, this requires continuous drug administration and may not generate complete skipping of the targeted exon. In this study, we apply genome editing with zinc finger nucleases (ZFNs) to permanently remove essential splicing sequences in exon 51 of the dystrophin gene and thereby exclude exon 51 from the resulting dystrophin transcript. This approach can restore the dystrophin reading frame in ~13% of DMD patient mutations. Transfection of two ZFNs targeted to sites flanking the exon 51 splice acceptor into DMD patient myoblasts led to deletion of this genomic sequence. A clonal population was isolated with this deletion and following differentiation we confirmed loss of exon 51 from the dystrophin mRNA transcript and restoration of dystrophin protein expression. Furthermore, transplantation of corrected cells into immunodeficient mice resulted in human dystrophin expression localized to the sarcolemmal membrane. Finally, we quantified ZFN toxicity in human cells and mutagenesis at predicted off-target sites. This study demonstrates a powerful method to restore the dystrophin reading frame and protein expression by permanently deleting exons.

## Introduction

Engineered site-specific nucleases have broadly enabled the precise manipulation of DNA sequences in complex genomes.^1^ The rapid development of designer enzymes such as zinc finger nucleases (ZFNs),^2,3^ transcription activator-like effector nucleases,^4^ and the more recently described RNA-guided CRISPR/Cas9 system^5^ has enabled the possibility of genome editing for gene therapy. Nuclease-mediated gene editing strategies create site-specific changes to the genome by generating targeted double-strand breaks that stimulate cellular DNA repair pathways. These pathways result either in error-prone DNA repair through nonhomologous end-joining or in specific changes guided by homology directed repair when co-delivered with a donor DNA repair template. Genome editing has been demonstrated to be a powerful method to study and/or correct monogenic mutations associated with hereditary disease.^6,7,8,9,10,11,12,13,14,15,16^

The severe X-linked hereditary disease Duchenne muscular dystrophy (DMD) is caused by mutations in the dystrophin gene^17^ that prematurely truncate this essential musculoskeletal protein. The loss of functional dystrophin expression causes progressive muscle wasting, typically leading to fatality by the third decade of life. Oligonucleotide-based exon skipping is a powerful method to exclude specific exons and has been exploited to restore dystrophin expression by removing exons adjacent to genomic deletions and restoring the normal reading frame.^18^ This strategy has predominantly been used to skip exon 51, which can address up to 13% of all DMD patient deletions.^19,20^ However, this transient restoration requires regular administration of the exon skipping drug for the duration of treatment. In contrast to this transient mRNA-targeted correction method, genome editing creates a stable change to the genome sequence of the cell that persists even after cell division. Targeted frameshifts using site-specific nucleases and the random small insertions and deletions (indels) that are generated during nonhomologous end-joining –based DNA repair have been used to correct the dystrophin gene with a single double-strand break.^12,21^ However, because the size of the indels is random, only approximately one-third of gene modifications will result in restoration of the correct reading frame. Furthermore, the introduction of random indels in the dystrophin gene results in heterogeneous changes to the final protein product that may impact the predictability, reliability, and immunogenicity of the resulting protein. Thus, there are distinct advantages to a gene correction method that results in a specific protein product with predictable functionality.

ZFNs are a widely studied tool to create targeted genetic modifications.^2,3^ ZFNs are polydactyl proteins that recognize DNA by linking individual zinc finger motifs in tandem, with each motif recognizing 3 bp of DNA. This array of zinc finger motifs is genetically fused to the catalytic domain of the FokI endonuclease to create a complete ZFN monomer.^22,23^ Site-specific double-strand breaks are created when two independent ZFN monomers bind to adjacent target DNA sequences on opposite strands in a head-to-head fashion, thereby permitting dimerization of FokI and cleavage of the target DNA. Several improvements have been made to enhance the specificity of these chimeric nucleases, including restriction of the spacer length between ZFN monomers,^24^ the engineering of obligate heterodimer FokI domains,^25,26,27^ the generation of autonomous ZFN pairs,^28^ and enhancement of the cleavage activity of FokI.^29^ In the past decade, numerous preclinical studies have described the utility of ZFNs to correct several human genetic mutations associated with sickle cell anemia,^13,14^ X-linked severe combined immunodeficiency,^8^ alpha-1-antitrypsin deficiency,^15^ and hemophilia.^9,16^ Significantly, ZFNs are now being tested in phase 1/2 clinical trials for disruption of the HIV-1 co-receptor CCR5.^30,31^

Genome editing can be utilized to generate precise genomic deletions at a targeted genomic locus.^28,32^ In this study, we engineered ZFNs to specifically delete exon 51 from the dystrophin gene to generate precise and reproducible frameshifts in the resulting transcript by the loss of this exon in DMD patient cells. The advantage of this method is that the resulting changes to the dystrophin transcript will generate restored dystrophin proteins with predictable protein sequence. First, we engineered a panel of ZFN proteins using the publicly available extended Modular Assembly (eMA)^33,34^ or Context-Dependent Assembly (CoDA)^35^ methods. Engineered nucleases were screened for activity by reporter assays in human cells and by monitoring gene editing activities at the intended chromosomal loci. Several ZFN pairs demonstrated measurable activity at their intended chromosomal target, including two ZFN pairs flanking the splice acceptor of exon 51. Active ZFN pairs were observed to have modest levels of cytotoxicity and one ZFN pair had low levels of detectable off-target mutagenesis. Two selected ZFN pairs were transfected into DMD patient cells, and a clonal cell line was isolated harboring the intended genomic deletion. After differentiation, we demonstrate that exon 51 is lost from the mRNA transcript, and dystrophin protein expression was restored. Furthermore, these cells express human dystrophin properly localized to the sarcolemma membrane following transplantation into the hind limb of immunodeficient mice. Importantly, this study demonstrates a general method to delete sequences from the genome that result in permanent exclusion of a specific exon from the resulting mRNA transcript, thereby predictably restoring expression of the dystrophin protein.

## Results

### Design of ZFNs targeted to exon 51

To identify ZFN pairs that are highly active, we created a large panel of ZFN pairs targeted across exon 51 of the dystrophin gene and its flanking introns with the goal of finding a combination of ZFN pairs to delete the entire exon or sequences important to its proper splicing in the resulting mRNA transcript (**Figure 1**, **Supplementary Tables **S**1** and **S2**). First, we engineered several ZFN pairs using a public webserver^36^ and the eMA method.^3,33,34^ The eMA approach is based on observations that ZFNs with four, five, or six zinc finger motifs in tandem are most likely to be highly active.^33^ Accordingly, we generated 45 engineered proteins consisting of three, four, five, and six zinc fingers for six different target sites using an established method of recombinant DNA assembly.^3,34^ An alternative method of ZFN design, CoDA, creates ZFNs with novel DNA recognition by recombining a library of previously characterized zinc finger arrays.^35^ We used a publicly available webserver^37^ to identify seven CoDA ZFN targets. All seven corresponding ZFN pairs were generated by gene synthesis. Together, these eMA and CoDA ZFN pairs are designed to flank the entire exon or either of the two splice junctions on the 5′ or 3′ end of exon 51. We predicted that deletion of one or more of these conserved splice junctions would result in loss of the entire exon from the dystrophin transcript.

### Screening for activity of eMA ZFNs

We screened 24 eMA ZFN pairs using the wild-type FokI domain for activity against their cognate target site to identify optimal zinc finger compositions using an episomal luciferase reporter assay.^38^ This assay utilizes a split luciferase gene that has a specific ZFN target site flanked by two identical regions of the luciferase gene that will recombine to form a complete luciferase gene when the target site is correctly recognized and cleaved by a ZFN pair (**Figure 2a**). Following transfection into human cells, nine candidate eMA ZFNs with significant levels (*P* < 0.01 compared to control) of activity at four distinct target sites were identified for further analysis (**Figure 2b**). Similar to previous studies of ZFNs generated by eMA,^33^ increased activity was observed as additional zinc finger motifs were added to a ZFN monomer, particularly for four or more zinc fingers per monomer. Two ZFN monomers, DZF-5 left monomer and DZF-6 left monomer, contained up to only 5 or 4 zinc fingers, respectively, because the longer targets contained triplets for which no motifs were available in the modular assembly library used to engineer these proteins.

### Evaluation of ZFN activity at endogenous targets

Since CoDA ZFNs have an established high success rate,^35^ all seven ZFN pairs were immediately tested for activity at chromosomal loci. Plasmids were assembled encoding the obligate heterodimeric FokI ELD/KKR domains^26^ containing enhanced Sharkey mutations^29^ fused to the nine highly active eMA ZFNs (**Figure 2b**) and seven designed CoDA ZFNs. The plasmids were electroporated into human DMD myoblasts to test their ability to cleave their chromosomal targets. Using the Surveyor assay,^39^ we identified three eMA ZFNs and three CoDA ZFNs that had detectable activity at the intended chromosomal locus (**Figure 3a**). This success rate of ~33% (3/9) of eMA ZFNs that were previously validated by the episomal reporter assay (**Figure 2b**) and 43% (3/7) of unvalidated CoDA ZFNs is comparable to previous studies exploring these approaches.^33,35^ Gene modification was still detectable for four of these six ZFN pairs after 10 days and remained stable (<25% signal change) for all three of the eMA ZFNs (**Figure 3b**), although the activity levels for several ZFNs were near the detection limit for this assay (~0.5–1%). Despite efficient gene editing activity at 3 days posttransfection, all three CoDA ZFNs showed a substantial or complete loss of signal by day 10 (**Figure 3b**), likely due to toxicity related to ZFN activity.

### Characterization of ZFN cytotoxicity

To further assess the toxicity of designed ZFNs, we transfected human cells with constructs carrying the six ZFNs with detectable chromosomal gene editing activity (**Figure 3a**,**c**). These experiments also used the obligate heterodimeric FokI ELD/KKR domains^26^ with enhanced Sharkey mutations^29^ to reduce the potential of homodimeric off-target activity. The cytotoxicity of these ZFNs was evaluated using a flow cytometry–based green fluorescent protein (GFP) retention assay that measures the survival of ZFN-transfected cells in a mixed population.^12,40^ All of the ZFNs tested had moderate levels of cytotoxicity that are significant compared to I-SceI (*P* < 0.0001), a known nontoxic nuclease. However, these cytotoxicity levels are within the extremes of two commonly used ZFNs targeting AAVS1 or CCR5 loci in human cells and significantly less cytotoxic than GZF3 (*P* < 0.0001), a known toxic ZFN pair^40^ (**Figure 3c**). Interestingly, a modest, but significant (*P* < 0.05), increase in cytotoxicity was observed as the number of ZF motifs was increased in the eMA ZFN pair targeted to the DZF-1 sequence. Despite displaying mild cytotoxicity, gene editing activity appeared stable for all DZF-1 targeting ZFN pairs (**Figure 3a**,**b**). Overall, the ZFN pairs engineered in this study had measured cytotoxicity comparable to two other well-characterized ZFNs targeting the AAVS1 (ref. 41) or CCR5 (ref. 31) loci.

### Restoration of the dystrophin gene by deleting exon 51 from the genome

Co-expression of two nucleases has been demonstrated to mediate deletion of the intervening chromosomal sequence between the two nuclease target sites.^28,32^ This could be exploited to permanently delete an exon at the genomic level, in contrast to current methods that transiently remove the exon at the mRNA level. To delete exon 51, we utilized two ZFNs, DZF-1 L6/R6 and DZF-9 with ELD/KKR- and Sharkey-modified FokI domains, that were identified to efficiently cleave chromosomal targets that flank the exon 51 splice acceptor sequence (**Figures 1**, **3a**, and **4a**). Co-expression of these ZFNs results in excision of the intervening 2.7 kb segment that is expected to contain sequences necessary to include exon 51 in the dystrophin mRNA transcript (**Figure 4a**). Plasmids encoding the DZF-1 L6/R6 and DZF-9 ZFN pairs were electroporated into Δ48–50 DMD patient myoblasts that are correctable by skipping of exon 51. When transfected separately, each ZFN pair showed significant levels of editing at the target locus by the Surveyor assay (**Figure 4b**). The expected genomic deletions were detected by PCR of genomic DNA only in cell populations treated with both ZFN pairs (**Figure 4c**). After verifying the presence of the expected genomic deletion, isogenic clones of DMD patient cells were derived and screened for this deletion event. One clone of interest was identified from ~500 screened clones and Sanger sequencing analysis confirmed a new junction of intron 50 and exon 51 sequences flanking the target sites of the ZFN pairs, resulting in the loss of the 2.7 kb region from the genome (**Figure 4d**). After this 2.7 kb sequence is removed, only a partial fragment of exon 51 remains in the genome. Since the deleted segment includes essential splice acceptor sequences, the remaining exon 51 fragment is unlikely to be incorporated into the dystrophin mRNA transcript, resulting in the loss of exon 51 entirely. After differentiating this clonal population into myoblasts, mRNA RT-PCR analysis showed that exon 51 was indeed efficiently removed from the dystrophin transcript (**Figure 4e**). Sanger sequencing of this PCR band showed the expected new junction of exons 47 and 52 (**Figure 4f**). Furthermore, genomic deletion and removal of exon 51 from the dystrophin transcript resulted in restored dystrophin expression in these cells (**Figure 4g**). These data demonstrate that gene editing is an effective method to specifically delete exons by removing essential splicing sequences from the genome.

### Human dystrophin expression *in vivo* following transplantation of genetically corrected cells

Transplantation of genetically corrected autologous myoblasts is a strategy to introduce functional dystrophin expression to skeletal muscle *in vivo*.^42^ To demonstrate the feasibility of this approach, we implanted into immunodeficient mice a clonally derived population of Δ48–50 DMD myoblasts with a corrected dystrophin gene carrying a deletion of exon 51 (**Figure 4d**–**g**), as well as untreated cells, and assessed human dystrophin expression *in vivo* (**Figure 5**). After 4 weeks, serial sections from two muscles for each condition were stained for human spectrin or dystrophin. Muscle fibers positive for human spectrin, which is expressed by both corrected and uncorrected engrafted human cells, were detected in cryosections of injected muscle tissue. Human dystrophin expression that co-localized with regions of human spectrin staining was detectable in muscles injected with genetically corrected cells. Notably, dystrophin was detected at the sarcolemma membrane, demonstrating proper protein localization in genetically corrected cells (**Figure 5** and **Supplementary Figure S1**). No fibers positive for human dystrophin were observed in sections from mice injected with the untreated DMD myoblasts (**Figure 5** and **Supplementary Figure S1**), indicating that the genetically corrected cells were the source of human dystrophin expression.

### Analysis of off-target genome editing

Off-target activity of engineered nucleases is a primary concern for gene editing therapies. To predict potential off-target sites, we utilized a publicly available tool, PROGNOS, that compiles and ranks potential off-target sites *in silico*.^43^ Using the ZFN2.0 detection algorithm in PROGNOS, we selected the top 10 potential off-target sites in the genome for both DZF-1 L6/R6 and DZF-9 ZFN pairs. Eight of the 10 identified off-target sites for each ZFN pair (**Supplementary Table S3**) were successfully amplified and assessed for activity by the Surveyor assay following transfection of the respective ZFN pairs that include the ELD/KKR- and Sharkey-modified obligate heterodimeric FokI^26,29^ into human DMD patient cells (**Figure 6**). DZF-1 L6/R6 had no observed off-target activity (0/8 loci, **Figure 6a**), while DZF-9 had measurable activity at 2/8 loci (**Figure 6b**), albeit at lower levels than the on-target locus. We further interrogated nuclease activity at these sites by deep sequencing, which has a lower limit of detection (**Table 1**, **Supplementary Table S4**). By this assay, the frequency of modified alleles was ~15% for both nucleases, slightly higher than measured by the Surveyor assay (**Figure 4b**). The deep sequencing also did not detect any activity at the eight off-target sites for DZF-1. The sequencing results also confirmed activity at the two DZF-9 off-target sites determined by the Surveyor assay, but also identified activity at a third off-target site (**Table 1**). In all cases, off-target activity was ≤25% of on-target activity. The frequency and size distributions of the indels were consistent with other reports of ZFN activity (**Supplementary Figure S2**). Notably, the three *bona fide* off-target loci with observable activity for the DZF-9 ZFN pair had substantial homology to the intended target site (1 mismatch, **Supplementary Table S3**). While we cannot rule out activity at the other off-target loci that may exist below the sensitivity of these assays or off-target activity at other loci that were not assessed here, these data demonstrate the relative specificity of our reagents that is comparable to other studies utilizing ZFNs for therapeutic applications.^9,31,44,45^

## Discussion

The rapid advancement of gene editing technologies has enabled precise correction of disease-related genes. This study introduces a novel method to correct the dystrophin gene by deleting exons from the genome, thereby permanently excluding the exons in the dystrophin transcript. Importantly, this genome editing method is compatible with many existing gene- and cell-based therapies in development for DMD. The implantation of autologous, genetically corrected myogenic cells is a widely explored strategy to introduce functional dystrophin expression *in vivo* that can persist for years following transplantation.^42^ Here, we demonstrate that a clonally derived, genetically corrected population of DMD patient cells can generate human dystrophin expression *in vivo* that is properly localized to the sarcolemma membrane. Similarly, this approach is compatible with gene correction of patient-derived induced pluripotent stem cells that can be subsequently clonally derived, characterized, and transplanted.^46,47,48^ Thus, gene correction by excising exons from the genome may be a viable method for creating an autologous population of corrected cells.

The utility of this approach will likely require enhancing the overall efficiency of exon deletion, particularly to expand this strategy to *in vivo* delivery of nucleases to correct the dystrophin gene *in situ*.^9,16^ It also may be possible to delete a shorter fragment than the 2.7 kb genomic region excised here, which would likely enhance the overall efficiency of this approach.^32^ Improving the activity and specificity of each nuclease pair may also further enhance deletion efficiency. All of the ZFNs engineered in this study displayed mild cytotoxicity in human cells similar to two benchmark ZFNs targeting AAVS1 and CCR5. One ZFN pair, DZF-9, had observable activity at 3 of 8 tested off-target loci in human cells. Notably, all eight of these target sites had only one mismatch to the intended target site. The fidelity of this approach may be further enhanced by incorporating orthogonal FokI obligate heterodimer mutations that would increase the specificity of this approach by limiting or eliminating unintended off-target pairings between monomers from each ZFN pair.^28^ This may reduce the potential for generating unintended chromosomal rearrangements by creating simultaneous double-strand breaks at unintended chromosomal loci.

Other gene editing technologies, such as CRISPR/Cas9 or transcription activator-like effector nucleases, are also alternatives to introduce similar genomic deletions with potentially increased efficiency, reduced toxicity, and/or off-target activity.^1,4,5^ However, the use of ZFNs in this study, in contrast to previous studies of editing the dystrophin gene with transcription activator-like effector nucleases^12^ or meganucleases,^7,21^ is notable in that ZFNs are already in use in clinical trials of *ex vivo* cell modification.^30^ Consequently, a path for translation of ZFN-based therapeutics has already been established. Additionally, the significantly smaller size of ZFNs allows for the packaging of a complete ZFN pair in a single AAV vector,^9,16^ which may be important for delivery and gene editing in skeletal and cardiac muscle *in vivo*.

Based on these results, deletion of genomic sequences containing splice sites for exon 51 results in the complete exclusion of the exon from the transcript and restoration of dystrophin protein expression. This study demonstrates a proof-of-principle approach to correcting the DMD reading frame with engineered nucleases by introducing predictable and repeatable changes to the genome that eliminate exon 51 from the transcript. Importantly, genome editing enables permanent changes in the corrected cell and its progeny and may enable correction of endogenous progenitor cells that can repopulate dystrophic tissue. This may be an advantage compared to other transient approaches to skip exon 51, although long-term expression of exon skipping constructs can also be achieved using viral gene transfer *in vivo*. In contrast to our previous study,^12^ the genetic changes here are intended to remove exon 51 and generate expression of a protein that is predictable based on the patient background deletion, similar to the changes caused by oligonucleotide-mediated exon skipping. The advantage of this method is that it reproducibly generates internally deleted proteins with known protein sequences and predictable functionality. This method presents a robust gene editing approach to restore the dystrophin gene that can be extended to address additional patient deletions common in DMD and serves as a blueprint for correcting the genetic basis of other monogenic hereditary disorders.

## Materials and Methods

***Plasmid constructs.*** eMA ZFNs were constructed using standard molecular biology techniques from a library of zinc finger modules with predefined specificity^3,34^ as described.^33^ In some cases, this library was supplemented with additional zinc finger domains targeting TGC or TCT by grafting a recognition helix sequence (**Supplementary Figure S2**) obtained from ZiFiT^16^ onto the Sp1C zinc finger motif backbone used by the other modular assembly zinc fingers. Coding regions for CoDA^35^ ZFNs were synthesized by BioBasic (Ontario, Canada) and cloned by standard molecular biology techniques. The linker used to join zinc finger domains to the FokI domain was dependent on the spacer size between the half-sites, with the amino acid sequences HLRGS for five base-pair spacers, HTGAAARA for six base-pair spacers, and HTGPGAAARA for seven base-pair spacers.^24^ For all episomal SSA assays, ZFNs with wild-type FokI domains were used. For all ZFN assays at chromosomal loci, FokI domains were modified using both the ELD/KKR obligate heterodimer mutations^26^ and the Sharkey mutations^29^ as described previously.^49^ All ZFN monomers were expressed from the CMV promoter on separate pcDNA3.1 plasmids (Invitrogen, Carlsbad, CA). Sequences for ZFN target sites and coding regions are provided in the **Supplementary Information**.

***Cell culture and transfection.*** HEK293T cells were obtained from the American Tissue Collection Center (Manassas, VA) through the Duke Cell Culture Facility and were maintained in Dulbecco's Modified Eagle's medium supplemented with 10% fetal bovine calf serum and 1% penicillin/streptomycin. Immortalized myoblasts^50^ from a wild-type patient or a DMD patient harboring a deletion of exons 48–50 (Δ48–50) in the dystrophin gene were maintained in skeletal muscle media (PromoCell, Heidelberg, Germany) supplemented with 20% fetal bovine calf serum (Sigma, St. Louis, MO), 50 μg/ml fetuin, 10 ng/ml human epidermal growth factor (Sigma), 1 ng/ml human basic fibroblast growth factor (Sigma), 10 μg/ml human insulin (Sigma), 1% GlutaMAX (Invitrogen), and 1% penicillin/streptomycin (Invitrogen). All cell lines were maintained at 37 °C and 5% CO_2_. Immortalized myoblasts were transfected with 10 µg of each expression vector by electroporation using the Gene Pulser XCell (Bio-Rad, Hercules, CA) with phosphate-buffered saline as an electroporation buffer using optimized conditions.^12^ Transfection efficiencies were measured by delivering an eGFP expression plasmid (pmaxGFP, Clontech, Mountain View, CA) and using flow cytometry. These efficiencies were routinely ≥95% for HEK293T and ≥70% for the immortalized myoblasts.

***Single-strand annealing assay.*** For this assay, eMA ZFNs were constructed in vectors utilizing the wild-type FokI domain. Construction of the SSA luciferase reporter plasmid pSSA Rep 3-1 has been described previously.^33,38^ Briefly, ZFN binding sites were introduced into the left and/or right arms of a split firefly luciferase gene by PCR and cloned into the *Bgl*II/*Eco*RI sites of the vector. All primers used for SSA construction are listed in the **Supplementary Table S5**. Human HEK293T cells were co-transfected with 25 ng of each ZFN monomer expression plasmid and 25 ng of SSA reporter plasmid in 96 well-plates using Lipofectamine 2000 (Invitrogen) according to the manufacturer's instructions. Cells were lysed directly in the plate and 30 µl of each lysate was transferred to 96-well plates for analysis using the Bright-Glo Luciferase Assay System (Promega E2620, Madison, WI) and a luminescence plate reader (1 second integration).

***Surveyor assay for endogenous gene modification.*** Genetic modifications were quantified using the Surveyor nuclease assay,^39^ which detects mutations characteristic of nuclease-mediated nonhomologous end-joining. After transfection, cells were incubated for 3 or 10 days at 37 °C, and genomic DNA was extracted using the DNeasy Blood and Tissue kit (Qiagen). The target locus was amplified by 35 cycles of PCR with the AccuPrime High Fidelity PCR kit (Invitrogen) using primers specific to each locus (**Supplementary Table S5**). The resulting PCR products were randomly melted and reannealed in a thermal cycler with the program: 95 °C for 240 seconds, followed by 85 °C for 60 seconds, 75 °C for 60 seconds, 65 °C for 60 seconds, 55 °C for 60 seconds, 45 °C for 60 seconds, 35 °C for 60 seconds, and 25 °C for 60 seconds with a −0.3 °C/second rate between steps. Following reannealing, 8 μl of PCR product was mixed with 1 μl of Surveyor Nuclease S and 1 μl of Enhancer S (Transgenomic) and incubated at 42 °C for 1 hour. After incubation, 6 μl of digestion product was loaded onto a 10% TBE polyacrylamide gel and run at 200 V for 30 minutes. The gels were stained with ethidium bromide and quantified by densitometry using the ImageLab software suite (Bio-Rad) as previously described.^12,39^

***PCR-based assay to detect genomic deletions.*** The exon 51 locus was amplified from genomic DNA by PCR (Invitrogen AccuPrime High Fidelity PCR kit) using Cel-I primers flanking the DZF-1 (CelI-DZF1/2/10-R) and DZF-9 (CelI-DZF9-F) target sites (**Supplementary Table S5**). PCR products were separated on TAE-agarose gels and stained with ethidium bromide for analysis.

***Clone isolation procedure.*** Immortalized DMD myoblasts were electroporated with 5 μg of each ZFN plasmid (10 μg total). After 7 days, isogenic clones were isolated by clonal density isolation. Briefly, treated DMD myoblasts were plated in 10 cm plates at 100–200 cells/plate and allowed to grow into small colonies (~7–14 days) before individual clones were isolated and transferred to 96-well plates for expansion and analysis. Genomic DNA was extracted from ~500 clones using the QuickExtract Kit (Epicentre, Madison, WI), and the target locus amplified by PCR using primers to detect the expected genomic deletion as above (**Supplementary Table S5**). The resulting PCR products were analyzed to identify a clone carrying the expected deletion and verified by Sanger sequencing.

***mRNA analysis.*** Immortalized myoblasts were differentiated into myofibers by replacing the growth medium with Dulbecco's Modified Eagle's medium supplemented with 1% insulin–transferrin–selenium (Invitrogen #51500056) and 1% penicillin/streptomycin (Invitrogen #15140) for 6 days before the cells were trypsinized and collected. Total RNA was isolated from these cells using the RNeasy Plus Mini Kit (QIAGEN) according to the manufacturer's instructions. RNA was reverse transcribed to cDNA using the VILO cDNA synthesis kit (Life Technologies #11754, Carlsbad, CA) and 1.5 µg of RNA for 2 hours at 42 °C according to the manufacturer's instructions. The target loci were amplified by 35 cycles of PCR with the AccuPrime High Fidelity PCR kit (Invitrogen) using primers annealing to exons 44 and 52 (**Supplementary Table S5**). PCR products were run on TAE-agarose gels and stained with ethidium bromide for analysis.

***Western blot analysis.*** To assess dystrophin protein expression, immortalized myoblasts were differentiated into myofibers as described above for 6 days. Cells were trypsinized, collected, and lysed in radioimmunoprecipitation assay buffer (Sigma) supplemented with a protease inhibitor cocktail (Sigma), and the total protein amount was quantified using the bicinchoninic acid assay according to the manufacturer's instructions (Pierce, Rockford, IL). Samples were then mixed with NuPAGE loading buffer (Invitrogen) and 5% β-mercaptoethanol and heated to 85 °C for 10 minutes. Twenty-five micrograms of protein were separated on 4–12% NuPAGE Bis-Tris gels (Invitrogen) with MES buffer (Invitrogen). Proteins were transferred to nitrocellulose membranes for 1–2 hours in 1× tris–glycine transfer buffer containing 10% methanol and 0.01% sodium dodecyl sulfate. The blot was then blocked for 1 hour with 5% milk-TBST at room temperature. Blots were probed with the following antibodies in 5% milk-TBST: anti-dystrophin C-terminus (1:25 overnight at 4 °C, Leica NCL-DYS2), anti-dystrophin rod domain (1:1,000 one hour at room temperature, Sigma MANDYS8), and anti-GAPDH (1:5,000 overnight at 4 °C, Cell Signal 2118S). Blots were then incubated with horseradish peroxidase–conjugated secondary antibodies (Santa Cruz, Dallas, TX) and visualized using the ChemiDoc chemilumescent system (Bio-Rad) and Western-C ECL substrate (Bio-Rad).

***Transplantation into immunodeficient mice.*** All animal experiments were conducted under protocols approved by the Duke Institutional Animal Care & Use Committee. Cells were trypsinized, collected, and washed in 1× Hank's Balanced Salt Solution (HBSS; Sigma). Two million cells were pelleted and resuspended in 5 µl 1× HBSS (Sigma) supplemented with cardiotoxin (Sigma #C9759) immediately prior to injection. These cells were transplanted into the hind limb tibialis anterior muscle of NOD.SCID.gamma (NSG) mice (Duke CCIF Breeding Core) by intramuscular injection using a single injection (*n* = 2 per condition). Four weeks after injection, mice were euthanized, and the tibialis anterior muscles were harvested. Two muscles for each condition were processed as serial sections for analysis, and representative sections for dystrophin or spectrin expression in approximate regions of each muscle section are shown.

***Immunofluorescence staining.*** Harvested tibialis anterior muscles were incubated in 30% glycerol overnight at 4 °C before mounting and freezing in optimal cutting temperature compound. Serial 10 µm sections were obtained by cryosectioning of the embedded muscle tissue at −20 °C. Cryosections were then washed in phosphate-buffered saline to remove the optimal cutting temperature compound and subsequently blocked for 30–60 minutes at room temperature in phosphate-buffered saline containing 10% heat-inactivated fetal bovine serum for spectrin detection or 5% heat-inactivated fetal bovine serum for dystrophin detection. Serial cryosections were incubated overnight at 4 °C with the following primary antibodies that are specific to human epitopes only: anti-spectrin (1:20, Leica NCL-SPEC1) or anti-dystrophin (1:10, Leica NCL-DYS3). After primary staining, spectrin or dystrophin expression was detected using a tyramide-based immunofluorescence signal amplification detection kit (Life Technologies; TSA Kit #22, catalog #T-20932). Briefly, cryosections were incubated with 1:200 goat antimouse biotin-XX secondary antibody (Life Technologies #B2763) in blocking buffer for 1 hour at room temperature. The signal was then amplified using streptavidin–horseradish peroxidase conjugates (1:100, from TSA Kit) in blocking buffer for 1 hour at room temperature. Finally, cryosections were incubated with tyramide-AlexaFluor488 conjugates (1:100, TSA kit) in manufacturer-provided amplification buffer for 10 minutes at room temperature. Stained cryosections were then mounted in ProLong AntiFade (Life Technologies #P36934) and visualized with conventional fluorescence microscopy.

***Cytotoxicity assay.*** To quantitatively assess nuclease-associated cytotoxicity, HEK293T cells were transfected with 10 ng of a GFP reporter and 100 ng of each ZFN expression vector using Lipofectamine 2000 according to the manufacturer's instructions (Invitrogen). The percentage of GFP-positive cells was assessed at 2 and 5 days by flow cytometry. The survival rate was calculated as the decrease in GFP-positive cells from days 2 to 5 and normalized to cells transfected with an empty nuclease expression vector as described previously.^40^

***Off-target analysis using the PROGNOS predictive algorithm.*** Potential off-target sites were identified using the recommended parameters and the ZFN2.0 alogrithm.^43^ Briefly, the maximum number of mismatches allowable by the PROGNOS server were considered for the length of the target site, heterodimeric and homodimeric target sites were allowed, and the top sites were binned for spacer lengths ideal for the zinc finger and FokI protein linker utilized in each monomer. For the purposes of this study, DZF-1 L6/R6 (linker: HTGAAARA) was assumed to have optimal activity on targets with 6 or 7 base-pair spacers between half-sites.^24^ Similarly, DZF-9 (linker: HLRGS) was assumed to have ideal activity on five or six base-pair spacers. Ten micrograms of each monomer was electroporated into human DMD patient myoblasts as described above, and genomic DNA was collected 3 days following transfection. Potential off-target loci were PCR amplified using primers generated from the PROGNOS output (**Supplementary Table S5**), and off-target activity was quantified using the Surveyor assay as described above.

***Deep sequencing.*** For DZF-1 and DZF-9, PCR was used to amplify both the target region as well as the eight candidate off-target sites for both ZFNs. PCR primers included Illumina (San Diego, CA) TruSeq sequencing primer sequences on the 5′ ends (**Supplementary Table S5**). A second round of PCR was then used to add Illumina flowcell binding sequences and experiment-specific indexes 5′ of the primer sequence. The resulting products were pooled and multiplex sequenced with 250 bp paired-end reads on an Illumina MiSeq instrument.

Because PCR products averaged 106 bp in length, the 3′ ends of paired reads overlapped, and that overlap was used to infer complete amplicon fragments via single ungapped alignment, parameterized to score each match as 5 and each mismatch as −4. Fragments were trimmed to remove Illumina adapter and primer sequences by performing ungapped alignment (parameterized as above to allow mismatches) of paired primer sequences to fragments and retaining only sequences between primers. Trimmed fragments were aligned to the human reference genome using BLAT. Any fragment with a top-scoring alignment that was different than the expected PCR product was discarded from downstream analysis. Each of the remaining fragments was then aligned to the reference genome sequence of the expected PCR product using a global affine alignment with the following parameterization: match = 5, mismatch = −4, gap open = −5, gap extend = −2. Alignments were then trimmed to the 20 bp flanking the middle of the ZFN target site; for some targets, the 20 bp window extended beyond the end of the alignment and was therefore truncated at the end of the alignment. Indel (insertion and/or deletion) statistics were gathered from these windows separately for each treatment/control following demultiplexing via sequenced barcodes, by counting gaps in the query and subject sequences of the resulting truncated alignments and tabulating numbers of fragments having any indels in these windows.

***Statistical analysis for SSA and cytotoxicity studies.*** Three independent experiments each consisting of two independent transfections were compiled as means and SEM (*n* = 6). Effects were evaluated with multivariate ANOVA and *post hoc* Student's *t*-test using JMP 10 Pro (JMP, Cary, NC).

**SUPPLEMENTARY MATERIAL** **Figure S1.** Additional immunofluorescence images probing human dystrophin expression. **Figure S2.** Distribution of indel size in DMD myoblasts treated with DZF-1 or DZF-9 as determined by deep sequencing. **Table S1.** Summary of target sites for ZFNs in this study. **Table S2.** Sequences of eMA zinc finger modules to supplement published Barbas modules. **Table S3.** PROGNOS ZFN v2.0 output. **Table S4.** Summary of deep sequencing data. **Table S5.** Primers used in this study. **Supplementary Information**

## Figures and Tables

**Figure 1:**
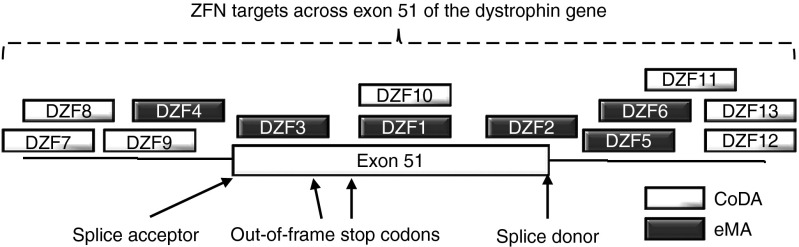
**Design of ZFNs targeted to exon 51**. ZFN pairs (shown as blocks) were designed as a panel of targets across exon 51 and the flanking introns. ZFN, zinc finger nuclease.

**Figure 2:**
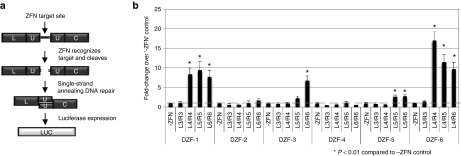
**Screening for active eMA ZFNs using an episomal reporter assay.** All ZFNs used the wild-type FokI nuclease domain. (**a**) Schematic of single-stranded annealing assay to detect ZFN activity. Each target site was cloned between a split luciferase reporter with flanking homology on either side of each target sequence. Luciferase expression occurs when a ZFN pair successfully recognizes and cleaves its cognate site in the reporter, causing single-strand annealing and recombination of an active luciferase gene. (**b**) Activity of different combinations of eMA ZFN pairs in HEK293T cells compared to cells transfected only with the reporter plasmid. eMA, extended Modular Assembly; ZFN, zinc finger nuclease.

**Figure 3:**
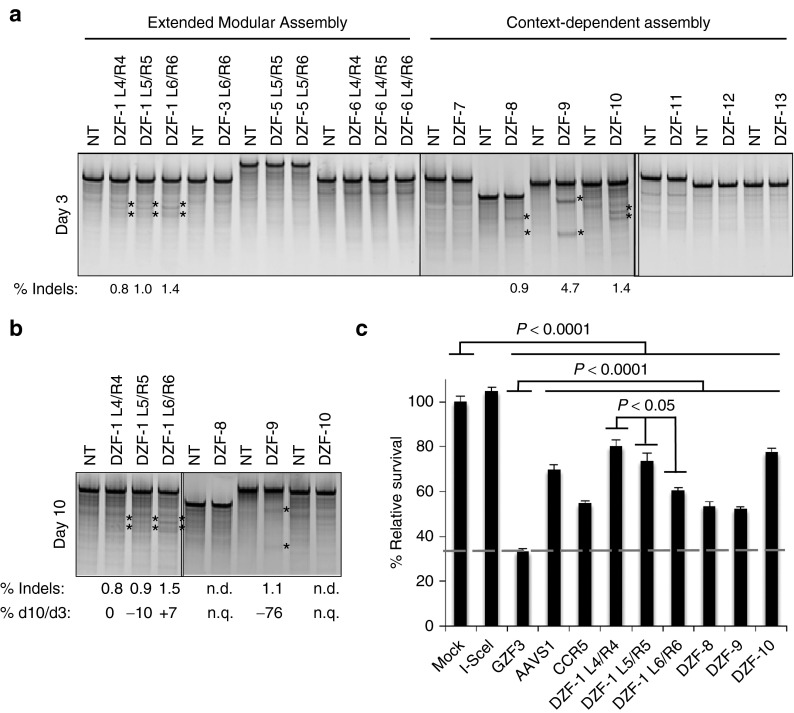
**Evaluation of selected ZFNs in human cells.** (**a**) All CoDA ZFNs and selected eMA ZFNs were transfected into wild-type myoblasts (10 μg of each monomer expression plasmid), and endogenous gene editing activity was measured at 3 days posttransfection by the Surveyor assay. (**b**) Activity of ZFN pairs that showed measurable activity in **a** was also determined at 10 days posttransfection to assess stability and survival of modified cells. The ratio of gene editing activity at 3 and 10 days was calculated from the data in **a** and **b**. n.d., not detected. n.q., not quantified. (**c**) Results of a cytotoxicity assay based on retention of GFP expression in HEK293T cells after transfection with the indicated nucleases and a GFP reporter. Percentage survival was calculated as the ratio of percent GFP-positive cells at days 2 and 5 posttransfection and normalized to transfection in the absence of nucleases. CoDA, Context-Dependent Assembly; eMA, extended Modular Assembly; GFP, green fluorescent protein; ZFN, zinc finger nuclease.

**Figure 4:**
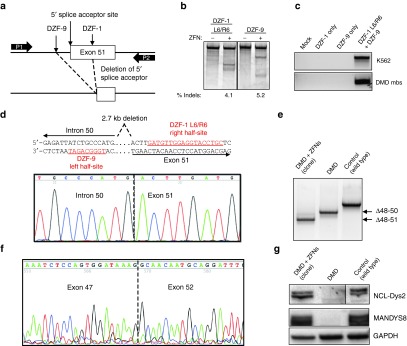
**Restoration of the dystrophin reading frame in DMD patient myoblasts.** (**a**) Schematic of strategy to delete exon 51 from the dystrophin gene locus. DZF-1 and DZF-9 flank the 5′ splice acceptor site of exon 51, which is removed after genomic deletion. P1/P2: primers used for detection of the genomic deletion by PCR in (**c**). (**b**) Gene modification activities of DZF-1 L6/R6 and DZF-9 as measured by the Surveyor assay 3 days after electroporation of 10 µg of each monomer expression cassette into DMD patient cells. (**c**) End-point genomic PCR across the deleted locus in human HEK293T or DMD myoblasts 3 days after treating cells with the indicated pair of nucleases. (**d**) Sanger sequencing of the PCR product from genomic DNA of a genetically corrected clonal cell population. Underlined sequences show target half-sites for the indicated ZFN target site. (**e**) End-point RT-PCR analysis of mRNA from control wild-type and untreated or a genetically corrected clonal population of DMD myoblasts after differentiation into myotubes. (**f**) Sanger sequencing of this PCR band showed the expected junction of exons 47 and 52. (**g**) Dystrophin expression as detected by western blot with antibodies to detect the C-terminus (NCL-DYS2) or rod domain (MANDYS8) in each of the indicated cell populations. All samples shown were run together on the same blot and cropped postimaging to remove extraneous lanes. However, different exposure times for the NCL-Dys2 western images were used to image DMD myoblasts and the genetically corrected clones compared to control samples to compensate for overexposure of control protein. The images for MANDYS8 and GAPDH are the same exposure time for all samples. DMD, Duchenne muscular dystrophy; ZFN, zinc finger nuclease.

**Figure 5:**
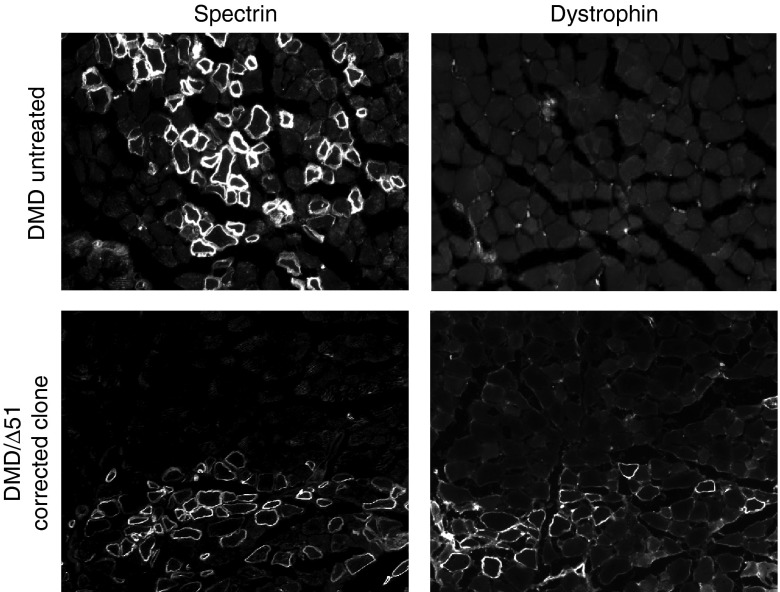
**Cell implantation and dystrophin expression *in vivo*.** Untreated or genetically corrected human Δ48–50 DMD myoblasts carrying a background deletion of exons 48–50 were injected into the hind limbs of immunodeficient mice and assessed for human-specific protein expression in muscle fibers after 4 weeks posttransplantation. Serial cryosections were stained with antihuman spectrin, which is expressed by both uncorrected and corrected myoblasts that have fused into mouse myofibers or anti-human dystrophin antibodies as indicated. DMD, Duchenne muscular dystrophy.

**Figure 6:**
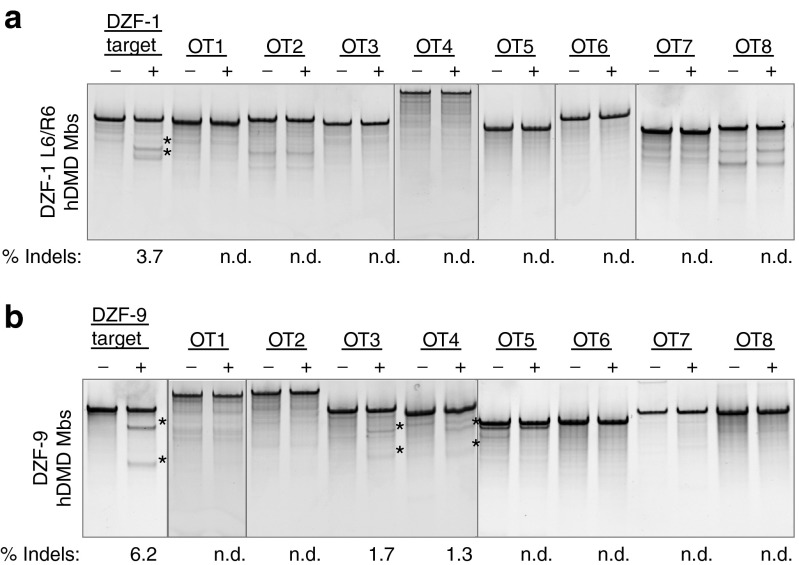
**Evaluation of ZFN off-target effects in human cells**. Human DMD myoblasts were electroporated with ten micrograms of DNA constructs encoding either DZF-1 L6/R6 or DZF-9. After 3 days, genomic DNA was analyzed by the Surveyor assay to measure activity at eight different potential off-target loci for (**a**) DZF-1 L6/R6 or (**b**) DZF-9 predicted by the PROGNOS algorithm.^43^ Asterisks indicate detectable Surveyor cleavage products. DMD, Duchenne muscular dystrophy; ZFN, zinc finger nuclease.

**Table 1:**
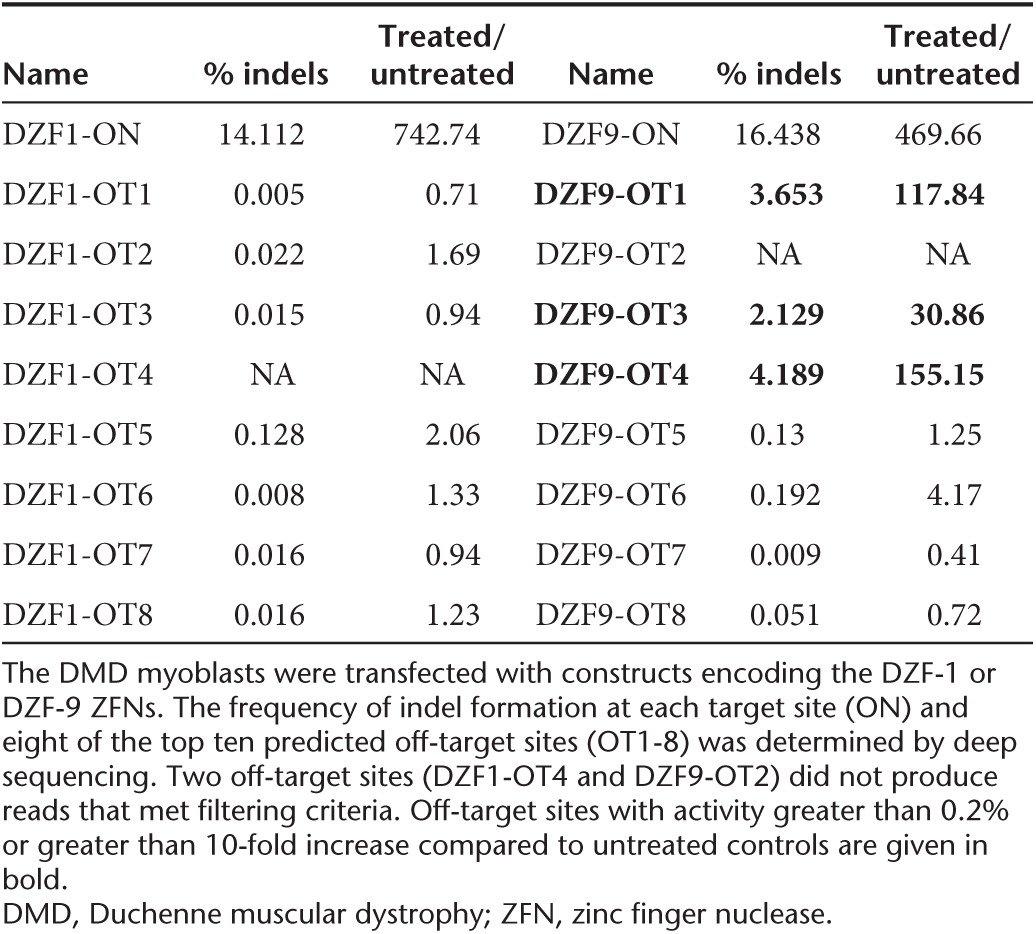
On-target and off-target gene editing activity by deep sequencing
