# Supplementary material for: Correction of Dystrophin Expression in Cells From Duchenne Muscular Dystrophy Patients Through Genomic Excision of Exon 51 by Zinc Finger Nucleases
Source: Mol Ther. 2015 Jan 13;23(3):523–32. doi: 10.1038/mt.2014.234 (PMC4351462; doi:10.1038/mt.2014.234)
Supplement: Supplementary Information [file mt2014234x1.pdf]

Correction of Dystrophin Expression in Cells from Duchenne Muscular Dystrophy Patients through Genomic Excision of Exon 51 by Zinc Finger Nucleases

David G. Ousterout[1], Ami M. Kabadi[1], Pratiksha I. Thakore[1], Pablo Perez-Pinera[1], Matthew T. Brown[1], William H. Majoros[4], Timothy E. Reddy[2,5], Charles A. Gersbach[1-3]

[1]Department of Biomedical Engineering, Duke University, Durham, North Carolina, United States of America, 27708

[2]Center for Genomic and Computational Biology, Duke University, Durham, North Carolina, United States of America, 27708

[3]Department of Orthopaedic Surgery, Duke University Medical Center, Durham, North Carolina, United States of America, 27710

[4]Program in Computational Biology and Bioinformatics, Duke University, Durham, North Carolina, United States of America, 27708

[5]Department of Biostatistics and Bioinformatics, Duke University Medical Center, Durham, North Carolina, United States of America, 27710

[*]Address for correspondence:
Charles A. Gersbach, Ph.D.
Department of Biomedical Engineering
Room 136 Hudson Hall, Box 90281
Duke University
Durham, NC 27708-0281
Phone: 919-613-2147
Fax: 919-668-0795
Email: charles.gersbach@duke.edu

Table of Contents

Supplementary Figure 1: Additional immunofluorescence images probing human dystrophin expression ……………………………………………………………………………….……..pg. 3

Supplementary Table 1: Sequences of ZFN target sites……………..………….……..…..pg. 4

Supplementary Table 2: Sequences of additional DNA recognition helices…………..…..pg. 4

Supplementary Table 3: PROGNOS ZFN v2.0 output……………………....…………pg. 5

Supplementary Table 4: Deep sequencing data for on and off-target sites…......…………pg. 6

Supplementary Figure 2: Distribution of indel sizes by deep sequencing………………....pg. 7

Supplementary Table 5: Sequences of primers used in this study……………………….....pg. 8

Supplementary sequences: Coding regions for all ZFNs in this study....…………………..pg. 11

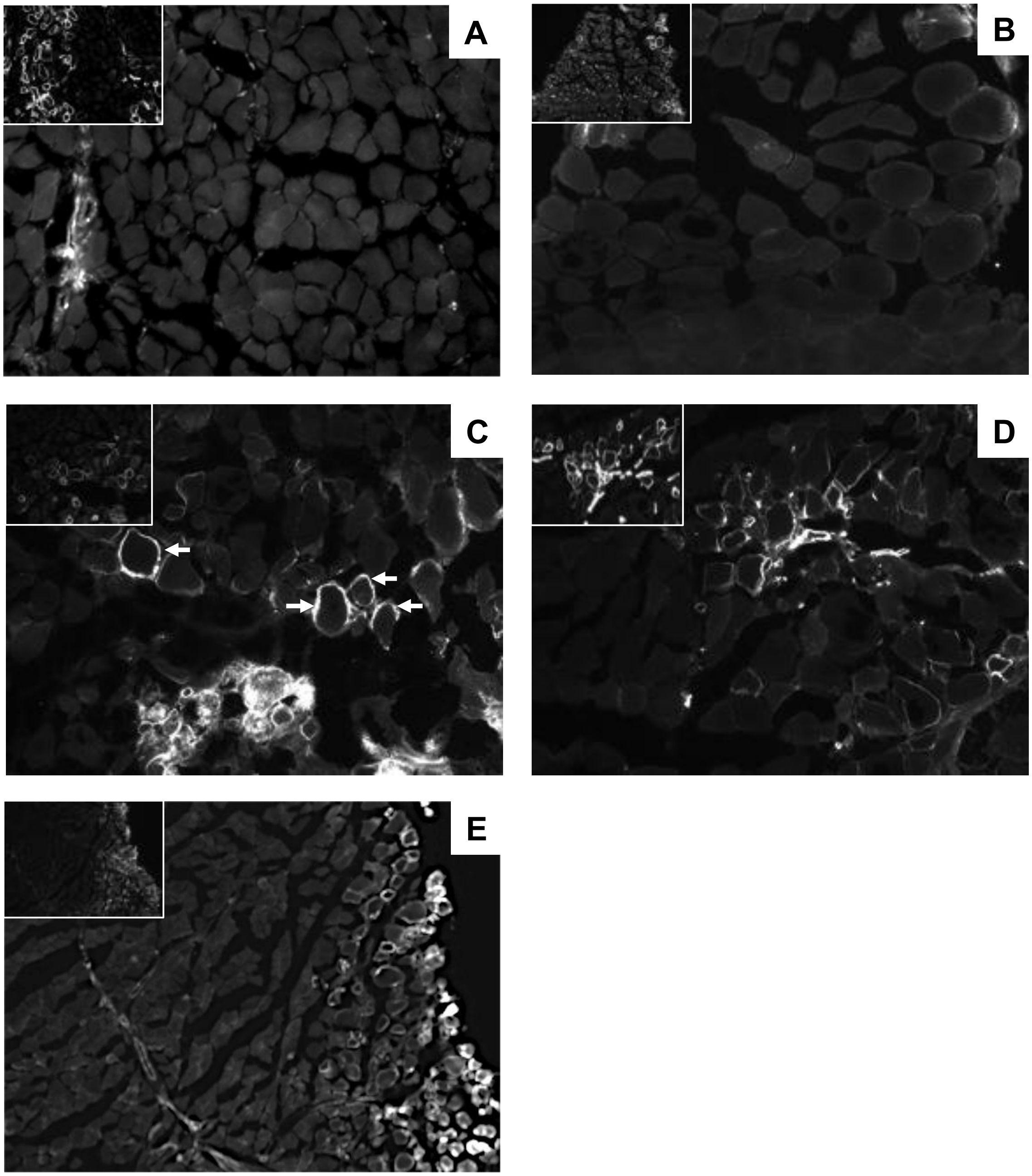

Supplementary Figure 1: Additional immunofluorescence images probing human dystrophin expression. Serial sections from regions stained with anti-human spectrin are shown inset in top left. (A-B) Sections from muscles injected with untreated human DMD myoblasts. (C-E) Sections from muscles injected with clonally derived DMD myoblasts carrying a deletion of exon 51 to that corrects the dystrophin reading frame. White arrows indicate examples of dystrophin positive fibers.

ZFN target	Target site	Spacer length (bp)
DZF-1	5'-CAA ACT AGA AAT GCC ATC TTCCTT GAT GTT GGA GGT ACC TGC<br>3'-GTT TGA TCT TTA CGG TAG AAGGAA CTA CAA CCT CCA TGG ACG	6
DZF-2	5'-ATG ATC ATC AAG CAG AAG GTATGA GAA AAA ATG ATA AAA GTT<br>3'-TAC TAG TAG TTC GTC TTC CATACT CTT TTT TAC TAT TTT CAA	6
DZF-3	5'-GAC TGT TAC TCT GGT GAC ACAACCT GTG GTT ACT AAG GAA ACT<br>3'-CTG ACA ATG AGA CCA CTG TGTTGGA CAC CAA TGA TTC CTT TGA	7
DZF-4	5'-CTT TAC CAC TTC CAC AAT GTATATG ATT GTT ACT GAG AAG GCT<br>3'-GAA ATG GTG AAG GTG TTA CATATAC TAA CAA TGA CTC TTC CGA	7
DZF-5	5'-CAC ATT CAC ATT CAC AATATA GTT ATG GAT ATG GAT GTA<br>3'-GAG TAA GTG TAA GTG TTATAT CAA TAC CTA TAC CTA CAT	6
DZF-6	5'-AAC TTC ACC AAT TCCATA GGA ATA AAA GTA ATT TGA<br>3'-TTG AAG TGG TTA AGGTAT CCT TAT TTT CAT TAA ACT	6
DZF-7	5'-AAC CCC ATC AAAAAGT GGG GGA AGG<br>3'-TTG GGG TAG TTTTTCA CCC CCT TCC	7
DZF-8	5'-ATC ATC TCC TCTGGTG GAT GAG GCT<br>3'-TAG TAG AGG AGACCAC CTA CTC CGA	7
DZF-9	5'-ATC TGC CCA TGACT GGC GCA GGG<br>3'-TAG ACG GGT ACTGA CCG CGT CCC	5
DZF-10	5'-GCC ATC TTC CTTGAT GTT GGA GGT<br>3'-CGG TAG AAG GAACTA CAA CCT CCA	6
DZF-11	5'-TGC TTC AGC CTCCTGA GTA GCT GGG<br>3'-ACG AAG TCG GAGGACT CAT CGA CCC	7
DZF-12	5'-GCC TCA GCC TCCCAAA GTG GTG GGA<br>3'-CGG AGT CGG AGGGTTT CAC CAC CCT	7
DZF-13	5'-CTC AGC CTC CCAAA GTG GTG GGA<br>3'-GAG TCG GAG GGTTT CAC CAC CCT	5

Supplementary Table 1: Summary of target sites for ZFNs in this study.

Triplet target	Helix sequence	Source
TGC	QRNALAG	ZiFiT F3 ID #631
TCT	QQRSLVG	ZiFiT F3 ID #790

Supplementary Table 2: Sequences of eMA zinc finger modules to supplement published Barbas modules.

**DZF-1 L6/R6**									
Top ten with spacers of 6-7 (optimal for this linker)									
**OT#**	**HS**	**Spacer**	**MM**	**Left Site**	**Right Site**	**Chr**	**Chr Region**	**Region**	**Gene**
1	L/R	6	5/4	CcAACTtGAgATGCCAgC	GgTGTgGGAGGTcgaTGC	chr12	121465217	Intron	OASL
2	L/L	7	6/2	CtAgCaAGAAgTcCCtTC	GATGGCATcTCTAcTTTG	chr2	164675298	Intergenic	-
3	L/L	7	4/3	CAAACTAtAAgTGCCgTg	GATGtCAaTTaTAGTTTG	chr6	3268596	Intergenic	-
4	L/R	6	6/6	attACTtctAATGCCATC	GATaTTGGAGaTAtaTtt	chr11	73441555	Intron	RAB6A
5	L/R	7	6/6	CAAgaacctAATGCCATC	GtcGgaGGAGGTACCccC	chr12	349901	Intron	SLC6A13
6	L/L	7	6/4	CAAAggAGAAATGCCAag	GATGGCtcTTaaAGTTgc	chr14	87103919	Intergenic	-
7	R/R	7	6/6	GCAaactCCTCCAACAag	GATGTTGGAaagACaaaC	chr8	141637370	Intron	EIF2C2
8	R/L	6	6/5	cCAGaTACCaCCttaATC	GcTGGCATgTCTcccTTG	chr1	220550165	Intergenic	-
**DZF-9**									
Top ten with spacers of 5-6 (optimal for this linker)									
**OT#**	**HS**	**Spacer**	**MM**	**Left Site**	**Right Site**	**Chr**	**Chr Region**	**Region**	**Gene**
1	R/L	6	1/0	CCCTGCcCC	TGGGCAGAT	chr6	12015411	Intron	HIVEP1
2	L/R	6	1/0	ATCTGCCCA	GGCGCAGtG	chr4	58293132	Intergenic	-
3	R/L	6	1/0	CCCTGCGCC	gGGGCAGAT	chr18	76473917	Intergenic	-
4	L/R	5	1/0	ATCTGCCCA	GGCGCAaGG	chr17	8113802	Intron	AURKB
5	L/R	5	1/0	ATCTGCCCA	GGCcCAGGG	chr7	128034911	Intron	IMPDH1
6	R/L	6	1/0	CCCTGgGCC	TGGGCAGAT	chr10	80912948	Intron	ZMIZ1
7	R/L	5	1/0	CCCTGaGCC	TGGGCAGAT	chr4	1595869	Intergenic	-
8	L/R	6	1/0	ATCTGCCCA	GGCtCAGGG	chr18	74383767	Intergenic	-

Supplementary Table 3: PROGNOS ZFN v2.0 output

	Untreated						Treated				
**Name**	**Indel Rate**	**Total Fragments**	**Deletion Rate**	**Insertion Rate**	**Mismatch Rate**		**Indel Rate**	**Total Fragments**	**Deletion Rate**	**Insertion Rate**	**Mismatch Rate**
**DZF1-ON**	0.00019	297376	0.00019	0.00011	0.0254		0.14112	275038	0.11332	0.02781	0.03581
**DZF1-OT1**	7.00E-05	392992	6.00E-05	2.00E-05	0.02629		5.00E-05	359847	3.00E-05	1.00E-05	0.02705
**DZF1-OT2**	0.00013	367911	0.00013	1.00E-05	0.0227		0.00022	304631	0.00022	3.00E-05	0.02474
**DZF1-OT3**	0.00016	306797	0.00016	0	0.02259		0.00015	242962	0.00015	2.00E-05	0.01974
**DZF1-OT4**	0	4	0	0	0		0	0	0	0	0
**DZF1-OT5**	0.00062	373972	0.00062	2.00E-05	0.02818		0.00128	384875	0.00127	3.00E-05	0.02746
**DZF1-OT6**	6.00E-05	382279	6.00E-05	1.00E-05	0.02677		8.00E-05	421802	8.00E-05	3.00E-05	0.02671
**DZF1-OT7**	0.00017	253294	0.00017	2.00E-05	0.02339		0.00016	385648	0.00016	1.00E-05	0.02519
**DZF1-OT8**	0.00013	331031	0.00013	0	0.01842		0.00016	374451	0.00016	3.00E-05	0.01869
**DZF9-ON**	0.00035	283889	0.00027	9.00E-05	0.02109		0.16438	247862	0.01432	0.15009	0.02759
**DZF9-OT1**	0.00031	368970	0.00027	0.00011	0.02059		0.03653	374476	0.00302	0.03352	0.01934
**DZF9-OT2**	0.33649	211	0.04739	0.30806	0.22275		0.0098	816	0	0.0098	0.02206
**DZF9-OT3**	0.00069	410574	0.00069	0	0.01957		0.02129	316583	0.00071	0.0206	0.01867
**DZF9-OT4**	0.00027	365973	0.00025	2.00E-05	0.02126		0.04189	396372	0.00222	0.0397	0.02002
**DZF9-OT5**	0.00104	406244	0.00104	3.00E-05	0.02447		0.0013	424538	0.0013	2.00E-05	0.02564
**DZF9-OT6**	0.00046	390369	0.00045	4.00E-05	0.01783		0.00192	422006	0.00052	0.00141	0.01933
**DZF9-OT7**	0.00022	54247	0.00022	0	0.01989		9.00E-05	90803	9.00E-05	0	0.02159
**DZF9-OT8**	0.00071	378717	0.0007	0.00059	0.9989		0.00051	380580	0.00051	0.00044	0.99877

Supplementary Table 4: Summary of deep sequencing data. The DMD myoblasts were transfected with constructs encoding the DZF-1 or DZF-9 ZFNs. The frequency of indel formation at each target site (ON, bold) and eight of the top ten predicted off-target sites (OT1-8) was determined by deep sequencing. Two off-target sites (DZF1-OT4 and DZF9-OT2) did not produce reads that met filtering criteria.

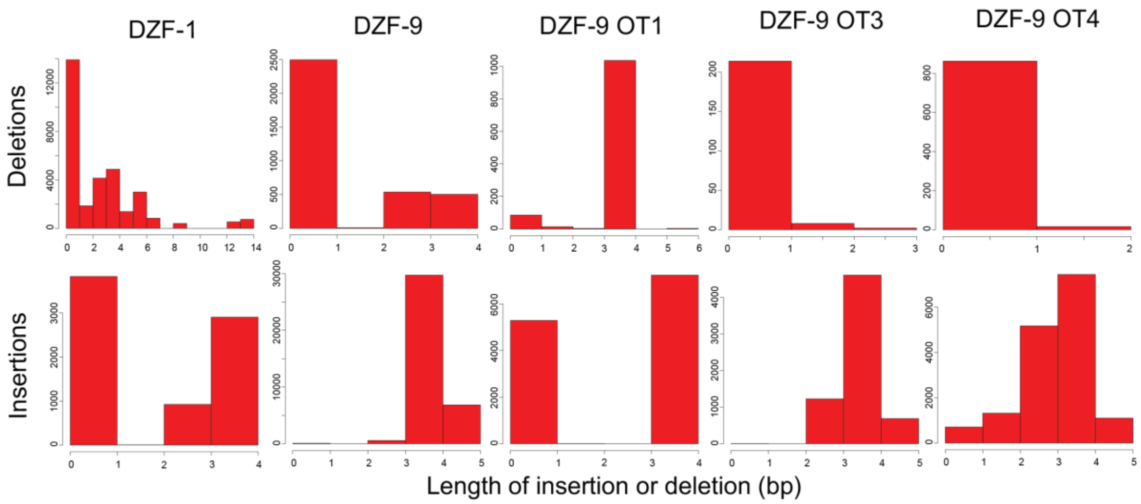

Supplementary Figure 2. Distribution of indel size in DMD myoblasts treated with DZF-1 or DZF-9 as determined by deep sequencing.

**Primer name**	**Primer sequence**	**Notes**
CelI-DZF-1/3/10-F	GAGTTTGGCTCAAATTGTTACTCTT	Forward Surveyor primer for DZF-1, DZF-3 and DZF-10
CelI-DZF-1/3/10-R	GGGAAATGGTCTAGGAGAGTAAAGT	Reverse Surveyor primer for DZF-1, DZF-3 and DZF-10
CelI-DZF-5-F	CCTCAGTGTAATCCATTTGGTAAAA	Forward Surveyor primer for DZF-5
CelI-DZF-6-F	CAAAGTTGTGCTGAAGGTATTTAGG	Forward Surveyor primer for DZF-6
CelI-DZF-5/6-R	CTGCTACTTACTGGGAATTTGACAT	Reverse Surveyor primer for DZF-5 and DZF-6
CelI-DZF-7-F	AACCATTGGAATTTACAGGATGAT	Forward Surveyor primer for DZF-7
CelI-DZF-7-R	GGCTGAGTTAAATGGTATTTCTGG	Reverse Surveyor primer for DZF-7
CelI-DZF-8-F	ACTTGCACCTCATTCTAATTGTGA	Forward Surveyor primer for DZF-8
CelI-DZF-8-R	CCTCCTACCTGAATGTTAGAGACAA	Reverse Surveyor primer for DZF-8
CelI-DZF-9-F	GATGCAAGAGATAGAGCAGTGAGA	Forward Surveyor primer for DZF-9
CelI-DZF-9-R	GTTTGGAAAAAGACAGAAAGGAAG	Reverse Surveyor primer for DZF-9
CelI-DZF-11-F	CCAATGACTTAAGGTTTCTTCACA	Forward Surveyor primer for DZF-11
CelI-DZF-11-R	CTGAATCATTGATGAAAAAGACCA	Reverse Surveyor primer for DZF-11
CelI-DZF-12/13-F	CCAACATGAGACTTTCTTTTTGTTT	Forward Surveyor primer for DZF-12 and DZF-13
CelI-DZF-12/13-R	AGCTGGAATATGCTTTTACTTTCC	Reverse Surveyor primer for DZF-12 and DZF-13
Dys-E44-F	TGGCGGCGTTTTCATTAT	Forward RT-PCR primer binding in exon 44
Dys-E52-R	TTCGATCCGTAATGATTGTTCTAGCC	Reverse RT-PCR primer binding in exon 52
SSA-fwd	CTAGCAAAATAGGCTGTCCC	Forward primer to construct SSA luciferase
SSA-luc-DZF-1-rev	GAGGAGGAATTCAGCAGGTACCTCCAACATCAAGGAAGATGGCATTTCTAGTTTGGTCACATAGGACCTCTCACACACAG	Reverse primer to construct SSA luciferase
SSA-luc-DZF-2-rev	GAGGAGGAATTCCAACTTTTATCATTTTTTCTCATACCTTCTGCTTGATGATCATCTCACATAGGACCTCTCACACACAG	Reverse primer to construct SSA luciferase
SSA-luc-DZF-3-rev	GAGGAGGAATTCCAGTTTCCTTAGTAACCACAGGTTGTGTCACCAGAGTAACAGTCTTCACATAGGACCTCTCACACACAG	Reverse primer to construct SSA luciferase
SSA-luc-DZF-4-rev	GAGGAGGAATTCAAGCCTTCTCAGTAACAATCATATACATTGTGGAAGTGGTAAAGATCACATAGGACCTCTCACACACAG	Reverse primer to construct SSA luciferase
SSA-luc-DZF-5-rev	GAGGAGGAATTCATACATCCATATCCATAACTATATTGTGAATGTGAATGTGTTCACATAGGACCTCTCACACACAG	Reverse primer to construct SSA luciferase
SSA-luc-DZF-6-rev	GAGGAGGAATTCATCAAATTACTTTTATTCCTATGGAATTGGTGAAGTTTTCACATAGGACCTCTCACACACAG	Reverse primer to construct SSA luciferase
DZF1-OT-1-F	CATGCTAGCTCCTACAAAGCACTG	Forward Surveyor primer for DZF-1 off-target site 1
DZF1-OT-1-R	GGGAAATGGGTACTGAAGAAGACG	Reverse Surveyor primer for DZF-1 off-target site 1
DZF1-OT-2-F	CTGTGCTGCCTATTGCTTTCTGTC	Forward Surveyor primer for DZF-1 off-target site 2
DZF1-OT-2-R	CTGGTTGTGTGCCTAGTGATGG	Reverse Surveyor primer for DZF-1 off-target site 2
DZF1-OT-3-F	CCCATTACTGCATTTGCGGTCTTG	Forward Surveyor primer for DZF-1 off-target site 3
DZF1-OT-3-R	TCAACCTTGCCTGCACGGAG	Reverse Surveyor primer for DZF-1 off-target site 3

DZF1-OT-4-F	CCTCTTCTCTTGGGATCTGTGAGT	Forward Surveyor primer for DZF-1 off-target site 4
DZF1-OT-4-R	GAGAACCCAATGTAATGTGTTCACTGAGC	Reverse Surveyor primer for DZF-1 off-target site 4
DZF1-OT-5-F	AAAGACACCTTTTCTGCCCTCACG	Forward Surveyor primer for DZF-1 off-target site 5
DZF1-OT-5-R	GTGCCCAGCCCAATTCTTTCTTGC	Reverse Surveyor primer for DZF-1 off-target site 5
DZF1-OT-6-F	GGTCCTGGTCCAAAGCAATTCTG	Forward Surveyor primer for DZF-1 off-target site 6
DZF1-OT-6-R	CGCCCGGCCAGATTTGTCTA	Reverse Surveyor primer for DZF-1 off-target site 6
DZF1-OT-7-F	CCACACACACAGGACACTGATC	Forward Surveyor primer for DZF-1 off-target site 7
DZF1-OT-7-R	CCAGAAGGCAGCCACTAGAAAC	Reverse Surveyor primer for DZF-1 off-target site 7
DZF1-OT-8-F	CTAGAATTACAGGCGTGAGCCACT	Forward Surveyor primer for DZF-1 off-target site 8
DZF1-OT-8-R	GCAGCTGAGTTGCAGGCATAAG	Reverse Surveyor primer for DZF-1 off-target site 8
DZF9-OT-1-F	ACGTTCCTGGGAAACACAGGG	Forward Surveyor primer for DZF-9 off-target site 1
DZF9-OT-1-R	CCACCAAAAGGCAGCTCCATAAAC	Reverse Surveyor primer for DZF-9 off-target site 1
DZF9-OT-2-F	GCACAGGGTACACACCCATTAAC	Forward Surveyor primer for DZF-9 off-target site 2
DZF9-OT-2-R	AGTCTCTCCATCCCCGAGGT	Reverse Surveyor primer for DZF-9 off-target site 2
DZF9-OT-3-F	CTGGTTTCTGCACCACATATTGCC	Forward Surveyor primer for DZF-9 off-target site 3
DZF9-OT-3-R	CACATGGCCGGCAGGAGAAA	Reverse Surveyor primer for DZF-9 off-target site 3
DZF9-OT-4-F	AAAAGGGAGCAGGTCAGCACAC	Forward Surveyor primer for DZF-9 off-target site 4
DZF9-OT-4-R	GGGGGAATTTGGGGAAACTTTCCT	Reverse Surveyor primer for DZF-9 off-target site 4
DZF9-OT-5-F	TGAGTCAGATGGCCCAGGGA	Forward Surveyor primer for DZF-9 off-target site 5
DZF9-OT-5-R	CTTGGAGCCTCCACAGGTGA	Reverse Surveyor primer for DZF-9 off-target site 5
DZF9-OT-6-F	TGGACTGAGGGAACCCCTCT	Forward Surveyor primer for DZF-9 off-target site 6
DZF9-OT-6-R	CAGATTCCCAGGGAAGCTCG	Reverse Surveyor primer for DZF-9 off-target site 6
DZF9-OT-7-F	GCGCGCTCGGGTGAAAAAATTAAG	Forward Surveyor primer for DZF-9 off-target site 7
DZF9-OT-7-R	TCCCCTTCTTCCACCTCCAG	Reverse Surveyor primer for DZF-9 off-target site 7
DZF9-OT-8-F	GGCTCCCCTCCTTGTTAATGTTG	Forward Surveyor primer for DZF-9 off-target site 8
DZF9-OT-8-R	AGTGAGGACGATGACCAGCG	Reverse Surveyor primer for DZF-9 off-target site 8
DZF1-ON-miseq-R	GTCTCGTGGGCTCGGAGATGTGTATAAGAGACAGag agaaagccagtcggtaagttct	Deep seq primers
DZF1-OT1-miseq-F	TCGTCGGCAGCGTCAGATGTGTATAAGAGACAGTAG GAAACAAGGAACAGGCTTCTA	Deep seq primers
DZF1-OT1-miseq-R	GTCTCGTGGGCTCGGAGATGTGTATAAGAGACAGTT TATGGATTGGCTGTATTAAGCA	Deep seq primers
DZF1-OT2-miseq-F	TCGTCGGCAGCGTCAGATGTGTATAAGAGACAGCTT TTTCCCGAAATTTTTACCTTT	Deep seq primers
DZF1-OT2-miseq-R	GTCTCGTGGGCTCGGAGATGTGTATAAGAGACAGTG AACTTCATGTTTGCTTGATTTT	Deep seq primers
DZF1-OT3-miseq-F	TCGTCGGCAGCGTCAGATGTGTATAAGAGACAGtgg tgggttctttttaggattaag	Deep seq primers
DZF1-OT3-miseq-R	GTCTCGTGGGCTCGGAGATGTGTATAAGAGACAGag aagggaagacgatgaaataaca	Deep seq primers
DZF1-OT4-miseq-F	TCGTCGGCAGCGTCAGATGTGTATAAGAGACAGgtc acagaatcctaggcttggaca	Deep seq primers
DZF1-OT4-miseq-R	GTCTCGTGGGCTCGGAGATGTGTATAAGAGACAGat	Deep seq primers

	catttgatttcttttgtctcttcc	
DZF1-OT5-miseq-F	TCGTCGGCAGCGTCAGATGTGTATAAGAGACAGAAACCAAATCAACAAACAGATGAA	Deep seq primers
DZF1-OT5-miseq-R	GTCTCGTGGGCTCGGAGATGTGTATAAGAGACAGTTTTCAAAGGGAAAGTAAACCTTG	Deep seq primers
DZF1-OT6-miseq-F	TCGTCGGCAGCGTCAGATGTGTATAAGAGACAGCACTCCATGGTTCTTATGTCAGAG	Deep seq primers
DZF1-OT6-miseq-R	GTCTCGTGGGCTCGGAGATGTGTATAAGAGACAGACTCTTCCTTGTCCCAGTAATGTC	Deep seq primers
DZF1-OT7-miseq-F	TCGTCGGCAGCGTCAGATGTGTATAAGAGACAGCACTATCAAAGCCAAAAAGAAACA	Deep seq primers
DZF1-OT7-miseq-R	GTCTCGTGGGCTCGGAGATGTGTATAAGAGACAGTTGTCAGGTTTGCCTTATTGAGTA	Deep seq primers
DZF1-OT8-miseq-F	TCGTCGGCAGCGTCAGATGTGTATAAGAGACAGatttaccaagtgcattcatctttt	Deep seq primers
DZF1-OT8-miseq-R	GTCTCGTGGGCTCGGAGATGTGTATAAGAGACAGcttttagagggatttttcttgctg	Deep seq primers
DZF9-ON-miseq-F	TCGTCGGCAGCGTCAGATGTGTATAAGAGACAGCAGATAGATGTTCTTCAGGTGGTG	Deep seq primers
DZF9-ON-miseq-R	GTCTCGTGGGCTCGGAGATGTGTATAAGAGACAGCTGAAGCATATTCAGAGGATGATG	Deep seq primers
DZF9-OT1-miseq-F	TCGTCGGCAGCGTCAGATGTGTATAAGAGACAGATAACTGGAGTAGCCACAGTAGCA	Deep seq primers
DZF9-OT1-miseq-R	GTCTCGTGGGCTCGGAGATGTGTATAAGAGACAGTAAAGTATCGCATCAGGCTACAAA	Deep seq primers
DZF9-OT2-miseq-F	TCGTCGGCAGCGTCAGATGTGTATAAGAGACAGTCTTTAGTAGAGACGGGGTTTCAC	Deep seq primers
DZF9-OT2-miseq-R	GTCTCGTGGGCTCGGAGATGTGTATAAGAGACAGACTGGTCTCGTGATCTGCCTAC	Deep seq primers
DZF9-OT3-miseq-F	TCGTCGGCAGCGTCAGATGTGTATAAGAGACAGTAAATAGGCTTTGCAGTACCTTCC	Deep seq primers
DZF9-OT3-miseq-R	GTCTCGTGGGCTCGGAGATGTGTATAAGAGACAGTTTTCACTGCTGACAGTTTTATCC	Deep seq primers
DZF9-OT4-miseq-F	TCGTCGGCAGCGTCAGATGTGTATAAGAGACAGctgctatcgtccctacctcctt	Deep seq primers
DZF9-OT4-miseq-R	GTCTCGTGGGCTCGGAGATGTGTATAAGAGACAGggagagtagcagtgccttggac	Deep seq primers
DZF9-OT5-miseq-F	TCGTCGGCAGCGTCAGATGTGTATAAGAGACAGTACTTAAGCCCTGTGCCTCAGTTT	Deep seq primers
DZF9-OT5-miseq-R	GTCTCGTGGGCTCGGAGATGTGTATAAGAGACAGAGCCAGAAACGATACTTCAGGTT	Deep seq primers
DZF9-OT6-miseq-F	TCGTCGGCAGCGTCAGATGTGTATAAGAGACAGatcctaggacttccctgacagc	Deep seq primers
DZF9-OT6-miseq-R	GTCTCGTGGGCTCGGAGATGTGTATAAGAGACAGctacaaggaggagggtccttattc	Deep seq primers
DZF9-OT7-miseq-F	TCGTCGGCAGCGTCAGATGTGTATAAGAGACAGccaagccggagcctcctctg	Deep seq primers
DZF9-OT7-miseq-R	GTCTCGTGGGCTCGGAGATGTGTATAAGAGACAGgtgacctgtgaatcggtttatct	Deep seq primers
DZF9-OT8-miseq-F	TCGTCGGCAGCGTCAGATGTGTATAAGAGACAGaagtgctacaaagagctccactg	Deep seq primers
DZF9-OT8-miseq-R	GTCTCGTGGGCTCGGAGATGTGTATAAGAGACAGcacctctgacaatatgaggatttg	Deep seq primers
Universal i5 primer	AATGATACGGCGACCACCGAGATCTACACTCGTCGGCAGCGTC	Universal primer for deep seq library
BC1	CAAGCAGAAGACGGCATACGAGATACATCGGTCTCGTGGGCTCGG	Barcode primer
BC2	CAAGCAGAAGACGGCATACGAGATTGGTCAGTCTCGTGGGCTCGG	Barcode primer
BC3	CAAGCAGAAGACGGCATACGAGATCACTGTGTCTCGTGGGCTCGG	Barcode primer
BC4	CAAGCAGAAGACGGCATACGAGATATTGGCGTCTCGTGGGCTCGG	Barcode primer

Supplementary Table 5: Primers used in this study

Supplementary sequences for ZFNs used in this study:

DZF-1 left ZFN

ATGGACTACAAAGACCATGACGGTGATTATAAAGATCATGACATCGATTACAAGGATGACGATGACAAGATGGCCCCCAA
GAAGAAGAGGAAGGTGGGCCGCCTCGAGCCCGGGGAAAAGCCATATAAATGCCCCGAGTGCGGCAAATCATTCAGCAGAA
AGGACGCCCTGAGAGGCCACCAGCGCACCCATACCGGGGAGAAACCTTACAAGTGCCCCGAGTGCGGCAAGAGTTTCAGC
CACAGGACCACCCTGACAAACCACCAGAGGACCCACACCGGGGAAAAGCCATATAAATGCCCCGAGTGCGGCAAATCATT
CAGCCAGAGAAACGCCCTGGCCGGCCACCAGCGCACCCATACCGGGGAAAAACCTTATAAGTGTCCCGAGTGCGGCAAGA
GTTTCAGTCACAAAAACGCACTTCAGAATCATCAGAGGACACATACCGGGGAAAAACCGTACAAGTGTCCTGAGTGCGGG
AAGAGTTTCTCCGATCCGGGCCACTTAGTAAGACATCAGAGGACACATACCGGGGAGAAGCCCTATAAATGTCCAGAATG
TGGAAAGTCCTTTAGCACGTCAGGAAACTTAGTAAGACACCAGCGAACTCATACCGGTGCGGCCGCCCGCGCCCTGGTGA
AGAGCGAGCTGGAGGAGAAGAAGTCCGAGCTGCGGCACAAGCTGAAGTACGTGCCCCACGAGTACATCGAGCTGATCGAG
ATCGCCAGGAACCCCACCCAGGACCGCATCCTGGAGATGAAGGTGATGGAGTTCTTCATGAAGGTGTACGGCTACAGGGG
AGAGCACCTGGGCGGAAGCAGAAAGCCTGACGGCGCCATCTATACAGTGGGCAGCCCCATCGATTACGGCGTGATCGTGG
ACACAAAGGCCTACAGCGGCGGCTACAATCTGCCTATCGGCCAGGCCGACGAGATGGAGAGATACGTGGAGGAGAACCAG
ACACGGGATAAGCACCTCAACCCCAACGAGTGGTGGAAGGTGTACCCTAGCAGCGTGACCGAGTTCAAGTTCCTGTTCGT
GAGCGGCCACTTCAAGGGCAACTACAAGGCCCAGCTGACCAGGCTGAACCACATCACCAACTGCAATGGCGCCGTGCTGA
GCGTGGAGGAGCTGCTGATCGGCGGCGAGATGATCAAAGCCGGCACCCTGACACTGGAGGAGGTGCGGCGCAAGTTCAAC
AACGGCGAGATCAACTTCTGA

DZF-1 right ZFN

ATGGACTACAAAGACCATGACGGTGATTATAAAGATCATGACATCGATTACAAGGATGACGATGACAAGATGGCCCCCAA
GAAGAAGAGGAAGGTGGGCCGCCTCGAGCCCGGGGAAAAGCCATATAAATGCCCCGAGTGCGGCAAATCATTCAGCCAGC
AGAGAAGCCTGGTGGGCCACCAGCGCACCCATACCGGGGAAAAGCCGTACAAATGTCCAGAGTGTGGAAAATCCTTTTCT
GATAAAAAGGACCTGACACGGCATCAGCGAACCCACACCGGGGAAAAGCCCTACAAGTGCCCGGAATGCGGCAAGTCTTT
TAGCACCAGCGGACATTTAGTAAGACACCAGAGAACCCACACCGGGGAGAAACCTTATAAATGCCCAGAATGCGGGAAAT
CGTTCAGTCAAAGAGCACATTTAGAAAGACATCAACGGACCCACACCGGGGAAAAGCCCTACAAGTGTCCTGAGTGCGGA
AAGTCTTTCTCCACTAGCGGTTCATTAGTAAGACACCAGAGGACACACACCGGGGAGAAGCCCTATAAATGTCCAGAATG
TGGAAAGTCCTTTAGCACGTCAGGGAACTTAGTAAGACACCAGCGAACTCATACCGGTGCGGCCGCCCGCGCCCTGGTGA
AGAGCGAGCTGGAGGAGAAGAAGTCCGAGCTGCGGCACAAGCTGAAGTACGTGCCCCACGAGTACATCGAGCTGATCGAG
ATCGCCAGGAACCCCACCCAGGACCGCATCCTGGAGATGAAGGTGATGGAGTTCTTCATGAAGGTGTACGGCTACAGGGG
AGAGCACCTGGGCGGAAGCAGAAAGCCTGACGGCGCCATCTATACAGTGGGCAGCCCCATCGATTACGGCGTGATCGTGG
ACACAAAGGCCTACAGCGGCGGCTACAATCTGCCTATCGGCCAGGCCGACGAGATGCAGAGATACGTGAAGGAGAACCAG
ACCCGGAATAAGCACATCAACCCCAACGAGTGGTGGAAGGTGTACCCTAGCAGCGTGACCGAGTTCAAGTTCCTGTTCGT
GAGCGGCCACTTCAAGGGCAACTACAAGGCCCAGCTGACCAGGCTGAACCGCAAAACCAACTGCAATGGCGCCGTGCTGA
GCGTGGAGGAGCTGCTGATCGGCGGCGAGATGATCAAAGCCGGCACCCTGACACTGGAGGAGGTGCGGCGCAAGTTCAAC
AACGGCGAGATCAACTTCTGA

DZF-2 left ZFN

ATGGACTACAAAGACCATGACGGTGATTATAAAGATCATGACATCGATTACAAGGATGACGATGACAAGATGGCCCCCAA
GAAGAAGAGGAAGGTGGGCCGCCTCGAGCCCGGGGAGAAGCCCTATAAATGCCCTGAATGTGGCAAGAGCTTCAGTACTA
GCGGGAATCTCACTGAACATCAGCGAACTCATACCGGGGAGAAGCCCTATAAATGTCCAGAATGTGGAAAGTCCTTTAGC
ACGTCAGGGAACTTAGTAAGACACCAGCGAACTCATACCGGGGAGAAGCCCTATAAATGTCCAGAATGTGGAAAGTCCTT
TAGCACGTCAGGGAACTTAGTAAGACACCAGCGAACTCATACCGGGGAAAAGCCCTATAAGTGTCCCGAATGCGGCAAGA
GTTTTAGTACTACTGGCGCACTCACAGAACACCAGCGCACTCACACCGGGGAAAAGCCTTATAAGTGCCCCGAATGCGGA
AAGAGCTTCTCAAGGAATGATGCACTTACCGAGCATCAAAGGACTCATACCGGGGAAAAGCCCTATAAGTGTCCCGAATG
CGGCAAGAGTTTTAGTACTACTGGCGCACTCACAGAACACCAGCGCACTCACACCGGTGCGGCCGCCCGCGCCCTGGTGA
AGAGCGAGCTGGAGGAGAAGAAGTCCGAGCTGCGGCACAAGCTGAAGTACGTGCCCCACGAGTACATCGAGCTGATCGAG
ATCGCCAGGAACCCCACCCAGGACCGCATCCTGGAGATGAAGGTGATGGAGTTCTTCATGAAGGTGTACGGCTACAGGGG
AGAGCACCTGGGCGGAAGCAGAAAGCCTGACGGCGCCATCTATACAGTGGGCAGCCCCATCGATTACGGCGTGATCGTGG
ACACAAAGGCCTACAGCGGCGGCTACAATCTGCCTATCGGCCAGGCCGACGAGATGGAGAGATACGTGGAGGAGAACCAG
ACACGGGATAAGCACCTCAACCCCAACGAGTGGTGGAAGGTGTACCCTAGCAGCGTGACCGAGTTCAAGTTCCTGTTCGT
GAGCGGCCACTTCAAGGGCAACTACAAGGCCCAGCTGACCAGGCTGAACCACATCACCAACTGCAATGGCGCCGTGCTGA
GCGTGGAGGAGCTGCTGATCGGCGGCGAGATGATCAAAGCCGGCACCCTGACACTGGAGGAGGTGCGGCGCAAGTTCAAC
AACGGCGAGATCAACTTCTGA

DZF-2 right ZFN

ATGGACTACAAAGACCATGACGGTGATTATAAAGATCATGACATCGATTACAAGGATGACGATGACAAGATGGCCCCCAA
GAAGAAGAGGAAGGTGGGCCGCCTCGAGCCCGGGGAAAAGCCCTACAAGTGTCCTGAGTGCGGAAAGTCTTTCTCCACTA
GCGGTTCATTAGTAAGACACCAGAGGACACACACCGGGGAGAAGCCATACAAATGCCCTGAGTGTGGAAAGTCATTTAGC
CAGCGAGCTAATCTGCGGGCCCACCAGCGGACCCACACCGGGGAGAAACCTTATAAGTGTCCTGAGTGCGGCAAAAGTTT
CTCTCAAAAGTCCTCCCTTATTGCCCATCAAAGGACCCATACCGGGGAGAAGCCCTATAAATGTCCCGAGTGCGGAAAGT
CCTTCTCACGGCGCGATGAATTGAACGTCCATCAGAGAACACACACCGGGGAGAAGCCATACAAATGCCCTGAGTGTGGA
AAGTCATTTAGCCAGCGAGCTAATCTGCGGGCCCACCAGCGGACCCACACCGGGGAAAAGCCATATAAATGCCCCGAGTG
CGGCAAATCATTCAGCCAAAGTAGCAACTTAGTAAGACACCAGCGCACCCATACCGGTGCGGCCGCCCGCGCCCTGGTGA
AGAGCGAGCTGGAGGAGAAGAAGTCCGAGCTGCGGCACAAGCTGAAGTACGTGCCCCACGAGTACATCGAGCTGATCGAG

ATCGCCAGGAACCCCACCCAGGACCGCATCCTGGAGATGAAGGTGATGGAGTTCTTCATGAAGGTGTACGGCTACAGGGG
AGAGCACCTGGGCGGAAGCAGAAAGCCTGACGGCGCCATCTATACAGTGGGCAGCCCCATCGATTACGGCGTGATCGTGG
ACACAAAGGCCTACAGCGGCGGCTACAATCTGCCTATCGGCCAGGCCGACGAGATGCAGAGATACGTGAAGGAGAACCAG
ACCCGGAATAAGCACATCAACCCCAACGAGTGGTGGAAGGTGTACCCTAGCAGCGTGACCGAGTTCAAGTTCCTGTTCGT
GAGCGGCCACTTCAAGGGCAACTACAAGGCCCAGCTGACCAGGCTGAACCGCAAAACCAACTGCAATGGCGCCGTGCTGA
GCGTGGAGGAGCTGCTGATCGGCGGCGAGATGATCAAAGCCGGCACCCTGACACTGGAGGAGGTGCGGCGCAAGTTCAAC
AACGGCGAGATCAACTTCTGA

DZF-3 left ZFN

ATGGACTACAAAGACCATGACGGTGATTATAAAGATCATGACATCGATTACAAGGATGACGATGACAAGATGGCCCCCAA
GAAGAAGAGGAAGGTGGGCCGCCTCGAGCCCGGGGAGAAACCATACAAATGCCCCGAGTGTGGAAAGTCATTTAGTGATC
CAGGCGCATTAGTAAGACATCAGCGGACACATACCGGGGAAAAGCCCTATAAATGTCCCGAGTGCGGAAAGTCTTTTTCC
AGCCCTGCCGACCTGACACGCCACCAACGAACGCACACCGGGGAAAAACCTTACAAGTGCCCTGAGTGCGGCAAGAGCTT
CTCTCAATCAAGTTCATTAGTAAGACACCAGAGGACTCATACCGGGGAAAAGCCCTATAAGTGCCCAGAGTGCGGGAAAT
CATTCTCACAGCTGGCACATCTTAGAGCCCACCAGCGGACCCACACCGGGGAAAAGCCGTACAAATGTCCAGAGTGTGGA
AAATCCTTTTCTGATAAAAAGGACCTGACACGGCATCAGCGAACCCACACCGGGGAGAAACCATACAAATGCCCCGAGTG
TGGAAAGTCATTTAGTGATCCAGGCGCATTAGTAAGACATCAGCGGACACATACCGGTCCCGGTGCGGCCGCCCGCGCCC
TGGTGAAGAGCGAGCTGGAGGAGAAGAAGTCCGAGCTGCGGCACAAGCTGAAGTACGTGCCCCACGAGTACATCGAGCTG
ATCGAGATCGCCAGGAACCCCACCCAGGACCGCATCCTGGAGATGAAGGTGATGGAGTTCTTCATGAAGGTGTACGGCTA
CAGGGGAGAGCACCTGGGCGGAAGCAGAAAGCCTGACGGCGCCATCTATACAGTGGGCAGCCCCATCGATTACGGCGTGA
TCGTGGACACAAAGGCCTACAGCGGCGGCTACAATCTGCCTATCGGCCAGGCCGACGAGATGGAGAGATACGTGGAGGAG
AACCAGACACGGGATAAGCACCTCAACCCCAACGAGTGGTGGAAGGTGTACCCTAGCAGCGTGACCGAGTTCAAGTTCCT
GTTCGTGAGCGGCCACTTCAAGGGCAACTACAAGGCCCAGCTGACCAGGCTGAACCACATCACCAACTGCAATGGCGCCG
TGCTGAGCGTGGAGGAGCTGCTGATCGGCGGCGAGATGATCAAAGCCGGCACCCTGACACTGGAGGAGGTGCGGCGCAAG
TTCAACAACGGCGAGATCAACTTCTGA

DZF-3 right ZFN

ATGGACTACAAAGACCATGACGGTGATTATAAAGATCATGACATCGATTACAAGGATGACGATGACAAGATGGCCCCCAA
GAAGAAGAGGAAGGTGGGCCGCCTCGAGCCCGGGGAAAAACCATACAAGTGCCCCGAGTGTGGCAAGAGCTTTAGTACCC
ACCTCGACCTGATTAGACACCAGCGCACCCACACCGGGGAAAAGCCATATAAATGCCCCGAGTGCGGCAAATCATTCAGC
CAAAGTAGCAACTTAGTAAGACACCAGCGCACCCATACCGGGGAAAAGCCATATAAGTGCCCTGAGTGTGGAAAGAGCTT
CAGTAGGAAGGATAACCTTAAAAACCACCAAAGAACCCACACCGGGGAAAAACCATACAAGTGCCCCGAGTGTGGCAAGA
GCTTTAGTACCCACCTCGACCTGATTAGACACCAGCGCACCCACACCGGGGAAAAGCCCTACAAGTGTCCTGAGTGCGGA
AAGTCTTTCTCCACTAGCGGTTCATTAGTAAGACACCAGAGGACACACACCGGGGAGAAGCCGTACAAGTGCCCTGAATG
TGGTAAGTCATTTTCGAGAAGTGATGAATTAGTAAGACACCAGCGGACTCATACCGGTCCCGGTGCGGCCGCCCGCGCCC
TGGTGAAGAGCGAGCTGGAGGAGAAGAAGTCCGAGCTGCGGCACAAGCTGAAGTACGTGCCCCACGAGTACATCGAGCTG
ATCGAGATCGCCAGGAACCCCACCCAGGACCGCATCCTGGAGATGAAGGTGATGGAGTTCTTCATGAAGGTGTACGGCTA
CAGGGGAGAGCACCTGGGCGGAAGCAGAAAGCCTGACGGCGCCATCTATACAGTGGGCAGCCCCATCGATTACGGCGTGA
TCGTGGACACAAAGGCCTACAGCGGCGGCTACAATCTGCCTATCGGCCAGGCCGACGAGATGCAGAGATACGTGAAGGAG
AACCAGACCCGGAATAAGCACATCAACCCCAACGAGTGGTGGAAGGTGTACCCTAGCAGCGTGACCGAGTTCAAGTTCCT
GTTCGTGAGCGGCCACTTCAAGGGCAACTACAAGGCCCAGCTGACCAGGCTGAACCGCAAAACCAACTGCAATGGCGCCG
TGCTGAGCGTGGAGGAGCTGCTGATCGGCGGCGAGATGATCAAAGCCGGCACCCTGACACTGGAGGAGGTGCGGCGCAAG
TTCAACAACGGCGAGATCAACTTCTGA

DZF-4 left ZFN

ATGGACTACAAAGACCATGACGGTGATTATAAAGATCATGACATCGATTACAAGGATGACGATGACAAGATGGCCCCCAA
GAAGAAGAGGAAGGTGGGCCGCCTCGAGCCCGGGGAAAAGCCATATAAGTGCCCTGAGTGTGGAAAGAGCTTCAGTAGGA
AGGATAACCTTAAAAACCACCAAAGAACCCACACCGGGGAAAAACCTTACAAGTGCCCTGAGTGCGGCAAGAGCTTCTCT
CAATCAAGTTCATTAGTAAGACACCAGAGGACTCATACCGGGGAGAAGCCGTACAAGTGCCCTGAATGTGGTAAGTCATT
TTCGAGAAGTGATGAATTAGTAAGACACCAGCGGACTCATACCGGGGAAAAGCCATATAAATGCCCCGAGTGCGGCAAAT
CATTCAGCCAAAGTAGCAACTTAGTAAGACACCAGCGCACCCATACCGGGGAGAAGCCGTACAAGTGCCCTGAATGTGGT
AAGTCATTTTCGAGAAGTGATGAATTAGTAAGACACCAGCGGACTCATACCGGGGAAAAACCTTATAAGTGTCCCGAGTG
CGGCAAGAGTTTCAGTCACAAAAACGCACTTCAGAATCATCAGAGGACACATACCGGTCCCGGTGCGGCCGCCCGCGCCC
TGGTGAAGAGCGAGCTGGAGGAGAAGAAGTCCGAGCTGCGGCACAAGCTGAAGTACGTGCCCCACGAGTACATCGAGCTG
ATCGAGATCGCCAGGAACCCCACCCAGGACCGCATCCTGGAGATGAAGGTGATGGAGTTCTTCATGAAGGTGTACGGCTA
CAGGGGAGAGCACCTGGGCGGAAGCAGAAAGCCTGACGGCGCCATCTATACAGTGGGCAGCCCCATCGATTACGGCGTGA
TCGTGGACACAAAGGCCTACAGCGGCGGCTACAATCTGCCTATCGGCCAGGCCGACGAGATGGAGAGATACGTGGAGGAG
AACCAGACACGGGATAAGCACCTCAACCCCAACGAGTGGTGGAAGGTGTACCCTAGCAGCGTGACCGAGTTCAAGTTCCT
GTTCGTGAGCGGCCACTTCAAGGGCAACTACAAGGCCCAGCTGACCAGGCTGAACCACATCACCAACTGCAATGGCGCCG
TGCTGAGCGTGGAGGAGCTGCTGATCGGCGGCGAGATGATCAAAGCCGGCACCCTGACACTGGAGGAGGTGCGGCGCAAG
TTCAACAACGGCGAGATCAACTTCTGA

DZF-4 right ZFN

ATGGACTACAAAGACCATGACGGTGATTATAAAGATCATGACATCGATTACAAGGATGACGATGACAAGATGGCCCCCAA
GAAGAAGAGGAAGGTGGGCCGCCTCGAGCCCGGGGAGAAACCATACAAGTGTCCAGAGTGCGGGAAAAGCTTTAGTACAA
GCGGTGAGTTAGTAAGACACCAACGAACACACACCGGGGAAAAGCCATATAAGTGCCCTGAGTGTGGAAAGAGCTTCAGT

AGGAAGGATAACCTTAAAAACCACCAAAGAACCCACACCGGGGAGAAGCCATATAAATGTCCCGAGTGTGGCAAGTCCTT
TTCTAGATCAGATAATTTAGTAAGACATCAGAGAACGCACACCGGGGAAAAACCATACAAGTGCCCCGAGTGTGGCAAGA
GCTTTAGTACCCACCTCGACCTGATTAGACACCAGCGCACCCACACCGGGGAAAAGCCCTACAAGTGTCCTGAGTGCGGA
AAGTCTTTCTCCACTAGCGGTTCATTAGTAAGACACCAGAGGACACACACCGGGGAAAAACCTTATAAGTGTCCCGAGTG
CGGCAAGAGTTTCAGTCACAAAAACGCACTTCAGAATCATCAGAGGACACATACCGGTCCCGGTGCGGCCGCCCGCGCCC
TGGTGAAGAGCGAGCTGGAGGAGAAGAAGTCCGAGCTGCGGCACAAGCTGAAGTACGTGCCCCACGAGTACATCGAGCTG
ATCGAGATCGCCAGGAACCCCACCCAGGACCGCATCCTGGAGATGAAGGTGATGGAGTTCTTCATGAAGGTGTACGGCTA
CAGGGGAGAGCACCTGGGCGGAAGCAGAAAGCCTGACGGCGCCATCTATACAGTGGGCAGCCCCATCGATTACGGCGTGA
TCGTGGACACAAAGGCCTACAGCGGCGGCTACAATCTGCCTATCGGCCAGGCCGACGAGATGCAGAGATACGTGAAGGAG
AACCAGACCCGGAATAAGCACATCAACCCCAACGAGTGGTGGAAGGTGTACCCTAGCAGCGTGACCGAGTTCAAGTTCCT
GTTCGTGAGCGGCCACTTCAAGGGCAACTACAAGGCCCAGCTGACCAGGCTGAACCGCAAAACCAACTGCAATGGCGCCG
TGCTGAGCGTGGAGGAGCTGCTGATCGGCGGCGAGATGATCAAAGCCGGCACCCTGACACTGGAGGAGGTGCGGCGCAAG
TTCAACAACGGCGAGATCAACTTCTGA

DZF-5 left ZFN

ATGGACTACAAAGACCATGACGGTGATTATAAAGATCATGACATCGATTACAAGGATGACGATGACAAGATGGCCCCCAA
GAAGAAGAGGAAGGTGGGCCGCCTCGAGCCCGGGGAGAAGCCATATAAATGTCCCGAGTGTGGCAAGTCCTTTTCTAGAT
CAGATAATTTAGTAAGACATCAGAGAACGCACACCGGGGAAAAGCCATATAAATGTCCCGAGTGCGGCAAATCCTTCTCT
ACCACTGGCAACCTCACAGTGCATCAACGGACTCACACCGGGGAGAAGCCGTACAAGTGCCCTGAATGTGGTAAGTCATT
TTCGAGAAGTGATGAATTAGTAAGACACCAGCGGACTCATACCGGGGAAAAGCCATATAAATGTCCCGAGTGCGGCAAAT
CCTTCTCTACCACTGGCAACCTCACAGTGCATCAACGGACTCACACCGGGGAGAAGCCGTACAAGTGCCCTGAATGTGGT
AAGTCATTTTCGAGAAGTGATGAATTAGTAAGACACCAGCGGACTCATACCGGTGCGGCCGCCCGCGCCCTGGTGAAGAG
CGAGCTGGAGGAGAAGAAGTCCGAGCTGCGGCACAAGCTGAAGTACGTGCCCCACGAGTACATCGAGCTGATCGAGATCG
CCAGGAACCCCACCCAGGACCGCATCCTGGAGATGAAGGTGATGGAGTTCTTCATGAAGGTGTACGGCTACAGGGGAGAG
CACCTGGGCGGAAGCAGAAAGCCTGACGGCGCCATCTATACAGTGGGCAGCCCCATCGATTACGGCGTGATCGTGGACAC
AAAGGCCTACAGCGGCGGCTACAATCTGCCTATCGGCCAGGCCGACGAGATGGAGAGATACGTGGAGGAGAACCAGACAC
GGGATAAGCACCTCAACCCCAACGAGTGGTGGAAGGTGTACCCTAGCAGCGTGACCGAGTTCAAGTTCCTGTTCGTGAGC
GGCCACTTCAAGGGCAACTACAAGGCCCAGCTGACCAGGCTGAACCACATCACCAACTGCAATGGCGCCGTGCTGAGCGT
GGAGGAGCTGCTGATCGGCGGCGAGATGATCAAAGCCGGCACCCTGACACTGGAGGAGGTGCGGCGCAAGTTCAACAACG
GCGAGATCAACTTCTGA

DZF-5 right ZFN

ATGGACTACAAAGACCATGACGGTGATTATAAAGATCATGACATCGATTACAAGGATGACGATGACAAGATGGCCCCCAA
GAAGAAGAGGAAGGTGGGCCGCCTCGAGCCCGGGGAAAAACCTTACAAGTGCCCTGAGTGCGGCAAGAGCTTCTCTCAAT
CAAGTTCATTAGTAAGACACCAGAGGACTCATACCGGGGAGAAGCCCTATAAATGTCCAGAATGTGGAAAGTCCTTTAGC
ACGTCAGGGAACTTAGTAAGACACCAGCGAACTCATACCGGGGAGAAGCCCTATAAATGTCCCGAGTGCGGAAAGTCCTT
CTCACGGCGCGATGAATTGAACGTCCATCAGAGAACACACACCGGGGAGAAGCCCTATAAATGTCCAGAATGTGGAAAGT
CCTTTAGCACGTCAGGGAACTTAGTAAGACACCAGCGAACTCATACCGGGGAGAAGCCCTATAAATGTCCCGAGTGCGGA
AAGTCCTTCTCACGGCGCGATGAATTGAACGTCCATCAGAGAACACACACCGGGGAAAAGCCCTACAAGTGTCCTGAGTG
CGGAAAGTCTTTCTCCACTAGCGGTTCATTAGTAAGACACCAGAGGACACACACCGGTGCGGCCGCCCGCGCCCTGGTGA
AGAGCGAGCTGGAGGAGAAGAAGTCCGAGCTGCGGCACAAGCTGAAGTACGTGCCCCACGAGTACATCGAGCTGATCGAG
ATCGCCAGGAACCCCACCCAGGACCGCATCCTGGAGATGAAGGTGATGGAGTTCTTCATGAAGGTGTACGGCTACAGGGG
AGAGCACCTGGGCGGAAGCAGAAAGCCTGACGGCGCCATCTATACAGTGGGCAGCCCCATCGATTACGGCGTGATCGTGG
ACACAAAGGCCTACAGCGGCGGCTACAATCTGCCTATCGGCCAGGCCGACGAGATGCAGAGATACGTGAAGGAGAACCAG
ACCCGGAATAAGCACATCAACCCCAACGAGTGGTGGAAGGTGTACCCTAGCAGCGTGACCGAGTTCAAGTTCCTGTTCGT
GAGCGGCCACTTCAAGGGCAACTACAAGGCCCAGCTGACCAGGCTGAACCGCAAAACCAACTGCAATGGCGCCGTGCTGA
GCGTGGAGGAGCTGCTGATCGGCGGCGAGATGATCAAAGCCGGCACCCTGACACTGGAGGAGGTGCGGCGCAAGTTCAAC
AACGGCGAGATCAACTTCTGA

DZF-6 left ZFN

ATGGACTACAAAGACCATGACGGTGATTATAAAGATCATGACATCGATTACAAGGATGACGATGACAAGATGGCCCCCAA
GAAGAAGAGGAAGGTGGGCCGCCTCGAGCCCGGGGAAAAGCCCTACAAGTGTCCTGAGTGCGGAAAGTCTTTCTCCACTA
GCGGTTCATTAGTAAGACACCAGAGGACACACACCGGGGAAAAGCCATATAAATGCCCCGAGTGCGGCAAATCATTCAGC
CAAAGTAGCAACTTAGTAAGACACCAGCGCACCCATACCGGGGAAAAGCCCTACAAGTGCCCGGAATGCGGCAAGTCTTT
TAGCACCAGCGGACATTTAGTAAGACACCAGAGAACCCACACCGGGGAAAAACCTTATAAGTGTCCCGAGTGCGGCAAGA
GTTTCAGTCACAAAAACGCACTTCAGAATCATCAGAGGACACATACCGGTGCGGCCGCCCGCGCCCTGGTGAAGAGCGAG
CTGGAGGAGAAGAAGTCCGAGCTGCGGCACAAGCTGAAGTACGTGCCCCACGAGTACATCGAGCTGATCGAGATCGCCAG
GAACCCCACCCAGGACCGCATCCTGGAGATGAAGGTGATGGAGTTCTTCATGAAGGTGTACGGCTACAGGGGAGAGCACC
TGGGCGGAAGCAGAAAGCCTGACGGCGCCATCTATACAGTGGGCAGCCCCATCGATTACGGCGTGATCGTGGACACAAAG
GCCTACAGCGGCGGCTACAATCTGCCTATCGGCCAGGCCGACGAGATGGAGAGATACGTGGAGGAGAACCAGACACGGGA
TAAGCACCTCAACCCCAACGAGTGGTGGAAGGTGTACCCTAGCAGCGTGACCGAGTTCAAGTTCCTGTTCGTGAGCGGCC
ACTTCAAGGGCAACTACAAGGCCCAGCTGACCAGGCTGAACCACATCACCAACTGCAATGGCGCCGTGCTGAGCGTGGAG
GAGCTGCTGATCGGCGGCGAGATGATCAAAGCCGGCACCCTGACACTGGAGGAGGTGCGGCGCAAGTTCAACAACGGCGA
GATCAACTTCTGA

DZF-6 right ZFN

ATGGACTACAAAGACCATGACGGTGATTATAAAGATCATGACATCGATTACAAGGATGACGATGACAAGATGGCCCCCAA
GAAGAAGAGGAAGGTGGGCCGCCTCGAGCCCGGGGAAAAGCCATATAAATGCCCCGAGTGCGGCAAATCATTCAGCCAGG
CCGGCCACCTGGCCAGCCACCAGCGCACCCATACCGGGGAAAAACCTTATAAGTGTCCCGAGTGCGGCAAGAGTTTCAGT
CACAAAAACGCACTTCAGAATCATCAGAGGACACATACCGGGGAAAAACCTTACAAGTGCCCTGAGTGCGGCAAGAGCTT
CTCTCAATCAAGTTCATTAGTAAGACACCAGAGGACTCATACCGGGGAGAAGCCATACAAATGCCCTGAGTGTGGAAAGT
CATTTAGCCAGCGAGCTAATCTGCGGGCCCACCAGCGGACCCACACCGGGGAGAAACCTTATAAGTGTCCTGAGTGCGGC
AAAAGTTTCTCTCAAAAGTCCTCCCTTATTGCCCATCAAAGGACCCATACCGGGGAGAAACCTTATAAATGCCCAGAATG
CGGGAAATCGTTCAGTCAAAGAGCACATTTAGAAAGACATCAACGGACCCACACCGGTGCGGCCGCCCGCGCCCTGGTGA
AGAGCGAGCTGGAGGAGAAGAAGTCCGAGCTGCGGCACAAGCTGAAGTACGTGCCCCACGAGTACATCGAGCTGATCGAG
ATCGCCAGGAACCCCACCCAGGACCGCATCCTGGAGATGAAGGTGATGGAGTTCTTCATGAAGGTGTACGGCTACAGGGG
AGAGCACCTGGGCGGAAGCAGAAAGCCTGACGGCGCCATCTATACAGTGGGCAGCCCCATCGATTACGGCGTGATCGTGG
ACACAAAGGCCTACAGCGGCGGCTACAATCTGCCTATCGGCCAGGCCGACGAGATGCAGAGATACGTGAAGGAGAACCAG
ACCCGGAATAAGCACATCAACCCCAACGAGTGGTGGAAGGTGTACCCTAGCAGCGTGACCGAGTTCAAGTTCCTGTTCGT
GAGCGGCCACTTCAAGGGCAACTACAAGGCCCAGCTGACCAGGCTGAACCGCAAAACCAACTGCAATGGCGCCGTGCTGA
GCGTGGAGGAGCTGCTGATCGGCGGCGAGATGATCAAAGCCGGCACCCTGACACTGGAGGAGGTGCGGCGCAAGTTCAAC
AACGGCGAGATCAACTTCTGA

DZF-7 left ZFN

ATGGACTACAAAGACCATGACGGTGATTATAAAGATCATGACATCGATTACAAGGATGACGATGACAAGATGGCCCCCAA
GAAGAAGAGGAAGGTGGGCCGCCTCGAGCCCGGGGAACGACCATTTCAGTGTAGGATCTGCATGCGAAATTTCTCCATTA
AGGCCATTTTGACACGCCACACAAGAACCCATACAGGCGAGAAACCGTTCCAGTGCAGGATATGCATGCGCAATTTCAGC
CGCAGAGAACACCTCGTCCGACACCTCCGGACACACACTGGCGAAAAGCCATTTCAATGCAGGATTTGCATGCGGAATTT
CAGCATCAGTCATAATCTCGCCAGGCACTTGAAAACACACACCGGTCCGGGAGCGGCCGCCCGCGCCCTGGTGAAGAGCG
AGCTGGAGGAGAAGAAGTCCGAGCTGCGGCACAAGCTGAAGTACGTGCCCCACGAGTACATCGAGCTGATCGAGATCGCC
AGGAACCCCACCCAGGACCGCATCCTGGAGATGAAGGTGATGGAGTTCTTCATGAAGGTGTACGGCTACAGGGGAGAGCA
CCTGGGCGGAAGCAGAAAGCCTGACGGCGCCATCTATACAGTGGGCAGCCCCATCGATTACGGCGTGATCGTGGACACAA
AGGCCTACAGCGGCGGCTACAATCTGCCTATCGGCCAGGCCGACGAGATGCAGAGATACGTGAAGGAGAACCAGACCCGG
AATAAGCACATCAACCCCAACGAGTGGTGGAAGGTGTACCCTAGCAGCGTGACCGAGTTCAAGTTCCTGTTCGTGAGCGG
CCACTTCAAGGGCAACTACAAGGCCCAGCTGACCAGGCTGAACCGCAAAACCAACTGCAATGGCGCCGTGCTGAGCGTGG
AGGAGCTGCTGATCGGCGGCGAGATGATCAAAGCCGGCACCCTGACACTGGAGGAGGTGCGGCGCAAGTTCAACAACGGC
GAGATCAACTTCTGA

DZF-7 right ZFN

ATGGACTACAAAGACCATGACGGTGATTATAAAGATCATGACATCGATTACAAGGATGACGATGACAAGATGGCCCCCAA
GAAGAAGAGGAAGGTGGGCCGCCTCGAGCCCGGGGAACGCCCATTCCAGTGCCGGATCTGTATGAGAAACTTCTCAAGGC
CCCACCACCTGGATGCCCATACAAGAACTCACACTGGAGAGAAGCCCTTCCAATGCCGGATTTGTATGAGGAATTTTAGC
CAGTCTGCCCATCTCAAACGCCATTTGAGGACCCACACGGGGGAGAAACCTTTTCAGTGCCGCATTTGCATGAGAAATTT
TTCCAGGACTGAGCACCTGGCCCGCCATCTGAAGACACATACCGGTCCGGGAGCGGCCGCCCGCGCCCTGGTGAAGAGCG
AGCTGGAGGAGAAGAAGTCCGAGCTGCGGCACAAGCTGAAGTACGTGCCCCACGAGTACATCGAGCTGATCGAGATCGCC
AGGAACCCCACCCAGGACCGCATCCTGGAGATGAAGGTGATGGAGTTCTTCATGAAGGTGTACGGCTACAGGGGAGAGCA
CCTGGGCGGAAGCAGAAAGCCTGACGGCGCCATCTATACAGTGGGCAGCCCCATCGATTACGGCGTGATCGTGGACACAA
AGGCCTACAGCGGCGGCTACAATCTGCCTATCGGCCAGGCCGACGAGATGGAGAGATACGTGGAGGAGAACCAGACACGG
GATAAGCACCTCAACCCCAACGAGTGGTGGAAGGTGTACCCTAGCAGCGTGACCGAGTTCAAGTTCCTGTTCGTGAGCGG
CCACTTCAAGGGCAACTACAAGGCCCAGCTGACCAGGCTGAACCACATCACCAACTGCAATGGCGCCGTGCTGAGCGTGG
AGGAGCTGCTGATCGGCGGCGAGATGATCAAAGCCGGCACCCTGACACTGGAGGAGGTGCGGCGCAAGTTCAACAACGGC
GAGATCAACTTCTGA

DZF-8 left ZFN

ATGGACTACAAAGACCATGACGGTGATTATAAAGATCATGACATCGATTACAAGGATGACGATGACAAGATGGCCCCCAA
GAAGAAGAGGAAGGTGGGCCGCCTCGAGCCCGGGGAACGCCCCTTCCAGTGTAGGATATGCATGAGAAACTTTTCCACCA
AACAGCGACTCGTGGTGCACACAAGGACACACACAGGGGAAAAACCTTTTCAGTGCAGAATTTGTATGCGGAATTTCTCT
GTGCGACATAACTTGACCAGGCACCTGCGAACACACACTGGAGAGAAACCCTTCCAATGTCGCATCTGCATGCGCAACTT
TTCTCAGACCACTCACCTTTCTCGCCACCTGAAGACGCATACCGGTCCAGGTGCGGCCGCCCGCGCCCTGGTGAAGAGCG
AGCTGGAGGAGAAGAAGTCCGAGCTGCGGCACAAGCTGAAGTACGTGCCCCACGAGTACATCGAGCTGATCGAGATCGCC
AGGAACCCCACCCAGGACCGCATCCTGGAGATGAAGGTGATGGAGTTCTTCATGAAGGTGTACGGCTACAGGGGAGAGCA
CCTGGGCGGAAGCAGAAAGCCTGACGGCGCCATCTATACAGTGGGCAGCCCCATCGATTACGGCGTGATCGTGGACACAA
AGGCCTACAGCGGCGGCTACAATCTGCCTATCGGCCAGGCCGACGAGATGGAGAGATACGTGGAGGAGAACCAGACACGG
GATAAGCACCTCAACCCCAACGAGTGGTGGAAGGTGTACCCTAGCAGCGTGACCGAGTTCAAGTTCCTGTTCGTGAGCGG
CCACTTCAAGGGCAACTACAAGGCCCAGCTGACCAGGCTGAACCACATCACCAACTGCAATGGCGCCGTGCTGAGCGTGG
AGGAGCTGCTGATCGGCGGCGAGATGATCAAAGCCGGCACCCTGACACTGGAGGAGGTGCGGCGCAAGTTCAACAACGGC
GAGATCAACTTCTGA

DZF-8 right ZFN

ATGGACTACAAAGACCATGACGGTGATTATAAAGATCATGACATCGATTACAAGGATGACGATGACAAGATGGCCCCCAA
GAAGAAGAGGAAGGTGGGCCGCCTCGAGCCCGGGGAAAGGCCATTCCAGTGCAGGATCTGTATGCGAAACTTCAGCAACA
AACAGGCACTCGACCGGCACACCAGGACCCACACAGGAGAAAAACCCTTTCAATGTCGAATATGTATGAGGAATTTCAGC

AGACAGGACAATCTGGGAAGGCACCTTCGCACTCATACCGGAGAGAAGCCCTTTCAGTGTCGGATTTGCATGCGGAACTT
CTCACTGAACTCCAACCTGGCCAGACACCTCAAGACTCACACCGGTCCAGGAGCGGCCGCCCGCGCCCTGGTGAAGAGCG
AGCTGGAGGAGAAGAAGTCCGAGCTGCGGCACAAGCTGAAGTACGTGCCCCACGAGTACATCGAGCTGATCGAGATCGCC
AGGAACCCCACCCAGGACCGCATCCTGGAGATGAAGGTGATGGAGTTCTTCATGAAGGTGTACGGCTACAGGGGAGAGCA
CCTGGGCGGAAGCAGAAAGCCTGACGGCGCCATCTATACAGTGGGCAGCCCCATCGATTACGGCGTGATCGTGGACACAA
AGGCCTACAGCGGCGGCTACAATCTGCCTATCGGCCAGGCCGACGAGATGCAGAGATACGTGAAGGAGAACCAGACCCGG
AATAAGCACATCAACCCCAACGAGTGGTGGAAGGTGTACCCTAGCAGCGTGACCGAGTTCAAGTTCCTGTTCGTGAGCGG
CCACTTCAAGGGCAACTACAAGGCCCAGCTGACCAGGCTGAACCGCAAAACCAACTGCAATGGCGCCGTGCTGAGCGTGG
AGGAGCTGCTGATCGGCGGCGAGATGATCAAAGCCGGCACCCTGACACTGGAGGAGGTGCGGCGCAAGTTCAACAACGGC
GAGATCAACTTCTGA

DZF-9 left ZFN

ATGGACTACAAAGACCATGACGGTGATTATAAAGATCATGACATCGATTACAAGGATGACGATGACAAGATGGCCCCCAA
GAAGAAGAGGAAGGTGGGCCGCCTCGAGCCCGGGGAAAGACCCTTTCAATGCAGAATCTGTATGCGAAACTTTAGTTCTA
AGCAAGCTCTCGCCGTGCATACCCGCACTCACACTGGTGAAAAGCCCTTCCAGTGTAGGATATGCATGCGCAACTTTTCA
CAGAGTACAACACTGAAGAGACACCTGCGCACCCACACGGGGGAAAAACCCTTTCAGTGCCGCATTTGTATGCGCAATTT
CTCCCGATCCGATCATCTCTCACTCCATCTGAAGACACACTTGCGGGGATCCCAGCTGGTGAAGAGCGAGCTGGAGGAGA
AGAAGTCCGAGCTGCGGCACAAGCTGAAGTACGTGCCCCACGAGTACATCGAGCTGATCGAGATCGCCAGGAACCCCACC
CAGGACCGCATCCTGGAGATGAAGGTGATGGAGTTCTTCATGAAGGTGTACGGCTACAGGGGAGAGCACCTGGGCGGAAG
CAGAAAGCCTGACGGCGCCATCTATACAGTGGGCAGCCCCATCGATTACGGCGTGATCGTGGACACAAAGGCCTACAGCG
GCGGCTACAATCTGCCTATCGGCCAGGCCGACGAGATGCAGAGATACGTGAAGGAGAACCAGACCCGGAATAAGCACATC
AACCCCAACGAGTGGTGGAAGGTGTACCCTAGCAGCGTGACCGAGTTCAAGTTCCTGTTCGTGAGCGGCCACTTCAAGGG
CAACTACAAGGCCCAGCTGACCAGGCTGAACCGCAAAACCAACTGCAATGGCGCCGTGCTGAGCGTGGAGGAGCTGCTGA
TCGGCGGCGAGATGATCAAAGCCGGCACCCTGACACTGGAGGAGGTGCGGCGCAAGTTCAACAACGGCGAGATCAACTTC
TGA

DZF-9 right ZFN

ATGGACTACAAAGACCATGACGGTGATTATAAAGATCATGACATCGATTACAAGGATGACGATGACAAGATGGCCCCCAA
GAAGAAGAGGAAGGTGGGCCGCCTCGAGCCCGGGGAGCGGCCTTTCCAATGCCGAATCTGCATGAGGAATTTCAGTCGCA
GGGCCCACCTGCAAAATCATACCCGGACCCATACCGGCGAGAAACCTTTTCAGTGTCGGATATGTATGAGGAACTTCTCT
CAGTCCACCACCCTGAAGCGGCACCTTCGCACACACACCGGGGAGAAGCCATTTCAATGTAGAATTTGCATGCGGAACTT
TTCTGATGGGGGTCACCTGACACGACACCTGAAGACGCATCTGAGGGGATCCCAGCTGGTGAAGAGCGAGCTGGAGGAGA
AGAAGTCCGAGCTGCGGCACAAGCTGAAGTACGTGCCCCACGAGTACATCGAGCTGATCGAGATCGCCAGGAACCCCACC
CAGGACCGCATCCTGGAGATGAAGGTGATGGAGTTCTTCATGAAGGTGTACGGCTACAGGGGAGAGCACCTGGGCGGAAG
CAGAAAGCCTGACGGCGCCATCTATACAGTGGGCAGCCCCATCGATTACGGCGTGATCGTGGACACAAAGGCCTACAGCG
GCGGCTACAATCTGCCTATCGGCCAGGCCGACGAGATGGAGAGATACGTGGAGGAGAACCAGACACGGGATAAGCACCTC
AACCCCAACGAGTGGTGGAAGGTGTACCCTAGCAGCGTGACCGAGTTCAAGTTCCTGTTCGTGAGCGGCCACTTCAAGGG
CAACTACAAGGCCCAGCTGACCAGGCTGAACCACATCACCAACTGCAATGGCGCCGTGCTGAGCGTGGAGGAGCTGCTGA
TCGGCGGCGAGATGATCAAAGCCGGCACCCTGACACTGGAGGAGGTGCGGCGCAAGTTCAACAACGGCGAGATCAACTTC
TGA

DZF-10 left ZFN

ATGGACTACAAAGACCATGACGGTGATTATAAAGATCATGACATCGATTACAAGGATGACGATGACAAGATGGCCCCCAA
GAAGAAGAGGAAGGTGGGCCGCCTCGAGCCCGGGGAACGACCTTTTCAATGTCGAATCTGTATGAGAAATTTCAGTTCCC
CGTCCAAGCTGGCCAGGCACACCAGGACGCATACCGGAGAAAAGCCCTTCCAATGCCGGATCTGCATGAGAAACTTTTCC
GTGAGACACAATCTGACAAGACATCTGAGGACGCACACCGGCGAGAAGCCCTTTCAATGCCGCATTTGTATGCGCAATTT
TTCCCAAAGAAATAACCTGGGGAGACACCTGAAGACTCATACCGGTGCGGCCGCCCGCGCCCTGGTGAAGAGCGAGCTGG
AGGAGAAGAAGTCCGAGCTGCGGCACAAGCTGAAGTACGTGCCCCACGAGTACATCGAGCTGATCGAGATCGCCAGGAAC
CCCACCCAGGACCGCATCCTGGAGATGAAGGTGATGGAGTTCTTCATGAAGGTGTACGGCTACAGGGGAGAGCACCTGGG
CGGAAGCAGAAAGCCTGACGGCGCCATCTATACAGTGGGCAGCCCCATCGATTACGGCGTGATCGTGGACACAAAGGCCT
ACAGCGGCGGCTACAATCTGCCTATCGGCCAGGCCGACGAGATGCAGAGATACGTGAAGGAGAACCAGACCCGGAATAAG
CACATCAACCCCAACGAGTGGTGGAAGGTGTACCCTAGCAGCGTGACCGAGTTCAAGTTCCTGTTCGTGAGCGGCCACTT
CAAGGGCAACTACAAGGCCCAGCTGACCAGGCTGAACCGCAAAACCAACTGCAATGGCGCCGTGCTGAGCGTGGAGGAGC
TGCTGATCGGCGGCGAGATGATCAAAGCCGGCACCCTGACACTGGAGGAGGTGCGGCGCAAGTTCAACAACGGCGAGATC
AACTTCTGA

DZF-10 right ZFN

ATGGACTACAAAGACCATGACGGTGATTATAAAGATCATGACATCGATTACAAGGATGACGATGACAAGATGGCCCCCAA
GAAGAAGAGGAAGGTGGGCCGCCTCGAGCCCGGGGAACGACCATTTCAGTGTAGAATATGCATGCGAAACTTCAGCATCC
CGAATCATCTGGCAAGACACACCCGGACCCATACCGGGGAAAAGCCGTTCCAATGTAGGATTTGCATGAGGAACTTTAGC
CAGTCAGCTCACCTCAAGCGGCACCTCCGGACACACACAGGGGAAAAACCCTTCCAGTGTCGCATATGTATGAGGAATTT
TTCACACCATAACAGTCTGACCCGCCATCTGAAGACCCACACCGGTGCGGCCGCCCGCGCCCTGGTGAAGAGCGAGCTGG
AGGAGAAGAAGTCCGAGCTGCGGCACAAGCTGAAGTACGTGCCCCACGAGTACATCGAGCTGATCGAGATCGCCAGGAAC
CCCACCCAGGACCGCATCCTGGAGATGAAGGTGATGGAGTTCTTCATGAAGGTGTACGGCTACAGGGGAGAGCACCTGGG
CGGAAGCAGAAAGCCTGACGGCGCCATCTATACAGTGGGCAGCCCCATCGATTACGGCGTGATCGTGGACACAAAGGCCT
ACAGCGGCGGCTACAATCTGCCTATCGGCCAGGCCGACGAGATGGAGAGATACGTGGAGGAGAACCAGACACGGGATAAG

CACCTCAACCCCAACGAGTGGTGGAAGGTGTACCCTAGCAGCGTGACCGAGTTCAAGTTCCTGTTCGTGAGCGGCCACTT
CAAGGGCAACTACAAGGCCCAGCTGACCAGGCTGAACCACATCACCAACTGCAATGGCGCCGTGCTGAGCGTGGAGGAGC
TGCTGATCGGCGGCGAGATGATCAAAGCCGGCACCCTGACACTGGAGGAGGTGCGGCGCAAGTTCAACAACGGCGAGATC
AACTTCTGAT

DZF-11 left ZFN

ATGGACTACAAAGACCATGACGGTGATTATAAAGATCATGACATCGATTACAAGGATGACGATGACAAGATGGCCCCCAA
GAAGAAGAGGAAGGTGGGCCGCCTCGAGCCCGGGGAGCGGCCGTTTCAATGCCGCATTTGTATGCGGAACTTTTCCCGCG
GTCAGGAGCTCAGGAGACACACAAGAACGCACACTGGCGAAAAACCCTTTCAGTGTAGGATCTGTATGAGGAATTTTTCA
CAACAAACAAACTTGACCCGCCACCTTAGAACTCACACTGGAGAGAAACCATTCCAATGTCGAATATGTATGCGCAACTT
CAGCGTGGGGAATAGCCTGACTCGGCATTTGAAGACCCATACCGGTCCCGGTGCGGCCGCCCGCGCCCTGGTGAAGAGCG
AGCTGGAGGAGAAGAAGTCCGAGCTGCGGCACAAGCTGAAGTACGTGCCCCACGAGTACATCGAGCTGATCGAGATCGCC
AGGAACCCCACCCAGGACCGCATCCTGGAGATGAAGGTGATGGAGTTCTTCATGAAGGTGTACGGCTACAGGGGAGAGCA
CCTGGGCGGAAGCAGAAAGCCTGACGGCGCCATCTATACAGTGGGCAGCCCCATCGATTACGGCGTGATCGTGGACACAA
AGGCCTACAGCGGCGGCTACAATCTGCCTATCGGCCAGGCCGACGAGATGGAGAGATACGTGGAGGAGAACCAGACACGG
GATAAGCACCTCAACCCCAACGAGTGGTGGAAGGTGTACCCTAGCAGCGTGACCGAGTTCAAGTTCCTGTTCGTGAGCGG
CCACTTCAAGGGCAACTACAAGGCCCAGCTGACCAGGCTGAACCACATCACCAACTGCAATGGCGCCGTGCTGAGCGTGG
AGGAGCTGCTGATCGGCGGCGAGATGATCAAAGCCGGCACCCTGACACTGGAGGAGGTGCGGCGCAAGTTCAACAACGGC
GAGATCAACTTCTGA

DZF-11 right ZFN

ATGGACTACAAAGACCATGACGGTGATTATAAAGATCATGACATCGATTACAAGGATGACGATGACAAGATGGCCCCCAA
GAAGAAGAGGAAGGTGGGCCGCCTCGAGCCCGGGGAAAGACCCTTCCAGTGTAGAATTTGCATGCGCAATTTTAGCAAGA
AGGACCATTTGCATAGACATACGCGAACCCATACTGGGGAGAAGCCTTTCCAGTGCCGCATCTGCATGCGGAATTTCAGC
CAGCGGTCCGATCTCACCCGACACCTGCGGACCCACACAGGTGAAAAGCCTTTTCAATGTCGGATATGCATGAGGAACTT
TAGTCAGAGCGGGACTCTCACTAGACATCTGAAGACGCACACCGGTCCCGGAGCGGCCGCCCGCGCCCTGGTGAAGAGCG
AGCTGGAGGAGAAGAAGTCCGAGCTGCGGCACAAGCTGAAGTACGTGCCCCACGAGTACATCGAGCTGATCGAGATCGCC
AGGAACCCCACCCAGGACCGCATCCTGGAGATGAAGGTGATGGAGTTCTTCATGAAGGTGTACGGCTACAGGGGAGAGCA
CCTGGGCGGAAGCAGAAAGCCTGACGGCGCCATCTATACAGTGGGCAGCCCCATCGATTACGGCGTGATCGTGGACACAA
AGGCCTACAGCGGCGGCTACAATCTGCCTATCGGCCAGGCCGACGAGATGCAGAGATACGTGAAGGAGAACCAGACCCGG
AATAAGCACATCAACCCCAACGAGTGGTGGAAGGTGTACCCTAGCAGCGTGACCGAGTTCAAGTTCCTGTTCGTGAGCGG
CCACTTCAAGGGCAACTACAAGGCCCAGCTGACCAGGCTGAACCGCAAAACCAACTGCAATGGCGCCGTGCTGAGCGTGG
AGGAGCTGCTGATCGGCGGCGAGATGATCAAAGCCGGCACCCTGACACTGGAGGAGGTGCGGCGCAAGTTCAACAACGGC
GAGATCAACTTCTGA

DZF-12 left ZFN

ATGGACTACAAAGACCATGACGGTGATTATAAAGATCATGACATCGATTACAAGGATGACGATGACAAGATGGCCCCCAA
GAAGAAGAGGAAGGTGGGCCGCCTCGAGCCCGGGGAGAGGCCGTTTCAATGTCGGATATGTATGAGAAACTTTTCTGCTC
CAAGCAAACTGAAACGGCACACACGGACTCACACCGGCGAGAAACCCTTTCAGTGTAGAATCTGTATGCGCAACTTCTCC
CAGAGAGAACACCTTACCACTCACTTGCGCACGCATACAGGCGAAAAGCCCTTTCAGTGCCGCATCTGCATGAGGAATTT
CTCAGAAAGCGGACATCTGAAGAGACACCTTAAAACACATACCGGTCCCGGAGCGGCCGCCCGCGCCCTGGTGAAGAGCG
AGCTGGAGGAGAAGAAGTCCGAGCTGCGGCACAAGCTGAAGTACGTGCCCCACGAGTACATCGAGCTGATCGAGATCGCC
AGGAACCCCACCCAGGACCGCATCCTGGAGATGAAGGTGATGGAGTTCTTCATGAAGGTGTACGGCTACAGGGGAGAGCA
CCTGGGCGGAAGCAGAAAGCCTGACGGCGCCATCTATACAGTGGGCAGCCCCATCGATTACGGCGTGATCGTGGACACAA
AGGCCTACAGCGGCGGCTACAATCTGCCTATCGGCCAGGCCGACGAGATGGAGAGATACGTGGAGGAGAACCAGACACGG
GATAAGCACCTCAACCCCAACGAGTGGTGGAAGGTGTACCCTAGCAGCGTGACCGAGTTCAAGTTCCTGTTCGTGAGCGG
CCACTTCAAGGGCAACTACAAGGCCCAGCTGACCAGGCTGAACCACATCACCAACTGCAATGGCGCCGTGCTGAGCGTGG
AGGAGCTGCTGATCGGCGGCGAGATGATCAAAGCCGGCACCCTGACACTGGAGGAGGTGCGGCGCAAGTTCAACAACGGC
GAGATCAACTTCTGA

DZF-12 right ZFN

ATGGACTACAAAGACCATGACGGTGATTATAAAGATCATGACATCGATTACAAGGATGACGATGACAAGATGGCCCCCAA
GAAGAAGAGGAAGGTGGGCCGCCTCGAGCCCGGGGAGCGACCATTCCAGTGTCGCATATGCATGCGGAACTTCAGTACAC
ATGCTCACTTGACCCGACATACACGAACACACACCGGAGAGAAACCTTTCCAGTGCAGGATCTGCATGAGAAATTTCAGC
CGAAGAGAAGTCCTGGAAAACCACCTGCGCACACATACCGGCGAAAAGCCGTTCCAGTGCCGCATTTGCATGAGGAACTT
CTCAAGGAAGGATGCTCTCCACGTCCACCTCAAGACGCACACCGGTCCGGGAGCGGCCGCCCGCGCCCTGGTGAAGAGCG
AGCTGGAGGAGAAGAAGTCCGAGCTGCGGCACAAGCTGAAGTACGTGCCCCACGAGTACATCGAGCTGATCGAGATCGCC
AGGAACCCCACCCAGGACCGCATCCTGGAGATGAAGGTGATGGAGTTCTTCATGAAGGTGTACGGCTACAGGGGAGAGCA
CCTGGGCGGAAGCAGAAAGCCTGACGGCGCCATCTATACAGTGGGCAGCCCCATCGATTACGGCGTGATCGTGGACACAA
AGGCCTACAGCGGCGGCTACAATCTGCCTATCGGCCAGGCCGACGAGATGCAGAGATACGTGAAGGAGAACCAGACCCGG
AATAAGCACATCAACCCCAACGAGTGGTGGAAGGTGTACCCTAGCAGCGTGACCGAGTTCAAGTTCCTGTTCGTGAGCGG
CCACTTCAAGGGCAACTACAAGGCCCAGCTGACCAGGCTGAACCGCAAAACCAACTGCAATGGCGCCGTGCTGAGCGTGG
AGGAGCTGCTGATCGGCGGCGAGATGATCAAAGCCGGCACCCTGACACTGGAGGAGGTGCGGCGCAAGTTCAACAACGGC
GAGATCAACTTCTGA

DZF-13 left ZFN

GCCATGGACTACAAAGACCATGACGGTGATTATAAAGATCATGACATCGATTACAAGGATGACGATGACAAGATGGCCCC
CAAGAAGAAGAGGAAGGTGGGCCGCCTCGAGCCCGGGGAGCGGCCTTTCCAGTGTCGGATCTGCATGCGGAATTTTTCTA
AACATTCAAACCTGACTAGGCATACGAGGACTCATACAGGAGAGAAGCCCTTCCAGTGTAGAATATGTATGCGAAACTTC
TCTCAGAGATCTGATTTGACGAGGCACTTGCGCACTCACACCGGCGAAAAACCTTTCCAATGCAGAATCTGCATGCGCAA
TTTCAGCCGGCAGGATAACCTTCAGCGGCACTTGAAGACACACCTGAGGGGATCCCAGCTGGTGAAGAGCGAGCTGGAGG
AGAAGAAGTCCGAGCTGCGGCACAAGCTGAAGTACGTGCCCCACGAGTACATCGAGCTGATCGAGATCGCCAGGAACCCC
ACCCAGGACCGCATCCTGGAGATGAAGGTGATGGAGTTCTTCATGAAGGTGTACGGCTACAGGGGAGAGCACCTGGGCGG
AAGCAGAAAGCCTGACGGCGCCATCTATACAGTGGGCAGCCCCATCGATTACGGCGTGATCGTGGACACAAAGGCCTACA
GCGGCGGCTACAATCTGCCTATCGGCCAGGCCGACGAGATGGAGAGATACGTGGAGGAGAACCAGACACGGGATAAGCAC
CTCAACCCCAACGAGTGGTGGAAGGTGTACCCTAGCAGCGTGACCGAGTTCAAGTTCCTGTTCGTGAGCGGCCACTTCAA
GGGCAACTACAAGGCCCAGCTGACCAGGCTGAACCACATCACCAACTGCAATGGCGCCGTGCTGAGCGTGGAGGAGCTGC
TGATCGGCGGCGAGATGATCAAAGCCGGCACCCTGACACTGGAGGAGGTGCGGCGCAAGTTCAACAACGGCGAGATCAAC
TTCTGA

DZF-13 right ZFN

ATGGACTACAAAGACCATGACGGTGATTATAAAGATCATGACATCGATTACAAGGATGACGATGACAAGATGGCCCCCAA
GAAGAAGAGGAAGGTGGGCCGCCTCGAGCCCGGGGAGCGACCATTCCAGTGTCGCATATGCATGCGGAACTTCAGTACAC
ATGCTCACTTGACCCGACATACACGAACACACACCGGAGAGAAACCTTTCCAGTGCAGGATCTGCATGAGAAATTTCAGC
CGAAGAGAAGTCCTGGAAAACCACCTGCGCACACATACCGGCGAAAAGCCGTTCCAGTGCCGCATTTGCATGAGGAACTT
CTCAAGGAAGGATGCTCTCCACGTCCACCTCAAGACGCACCTGAGGGGATCCCAGCTGGTGAAGAGCGAGCTGGAGGAGA
AGAAGTCCGAGCTGCGGCACAAGCTGAAGTACGTGCCCCACGAGTACATCGAGCTGATCGAGATCGCCAGGAACCCCACC
CAGGACCGCATCCTGGAGATGAAGGTGATGGAGTTCTTCATGAAGGTGTACGGCTACAGGGGAGAGCACCTGGGCGGAAG
CAGAAAGCCTGACGGCGCCATCTATACAGTGGGCAGCCCCATCGATTACGGCGTGATCGTGGACACAAAGGCCTACAGCG
GCGGCTACAATCTGCCTATCGGCCAGGCCGACGAGATGCAGAGATACGTGAAGGAGAACCAGACCCGGAATAAGCACATC
AACCCCAACGAGTGGTGGAAGGTGTACCCTAGCAGCGTGACCGAGTTCAAGTTCCTGTTCGTGAGCGGCCACTTCAAGGG
CAACTACAAGGCCCAGCTGACCAGGCTGAACCGCAAAACCAACTGCAATGGCGCCGTGCTGAGCGTGGAGGAGCTGCTGA
TCGGCGGCGAGATGATCAAAGCCGGCACCCTGACACTGGAGGAGGTGCGGCGCAAGTTCAACAACGGCGAGATCAACTTC
TGA
